# Fibronectin in cell adhesion and migration via N-glycosylation

**DOI:** 10.18632/oncotarget.19969

**Published:** 2017-08-07

**Authors:** Cheng-Te Hsiao, Hung-Wei Cheng, Chi-Ming Huang, Hao-Ru Li, Meng-Hsin Ou, Jie-Rong Huang, Kay-Hooi Khoo, Helen Wenshin Yu, Yin-Quan Chen, Yang-Kao Wang, Arthur Chiou, Jean-Cheng Kuo

**Affiliations:** ^1^ Institute of Biochemical Sciences, National Taiwan University, Taipei 10617, Taiwan; ^2^ Institute of Biological Chemistry, Academia Sinica, Taipei 115, Taiwan; ^3^ Institute of Biochemistry and Molecular Biology, National Yang-Ming University, Taipei 11221, Taiwan; ^4^ Biophotonics and Molecular Imaging Research Center, National Yang-Ming University, Taipei 11221, Taiwan; ^5^ Department of Cell Biology and Anatomy, National Cheng Kung University, Tainan 70101, Taiwan; ^6^ Institute of Biophotonics, National Yang-Ming University, Taipei 11221, Taiwan; ^7^ Proteomics Research Center, National Yang-Ming University, Taipei 11221, Taiwan

**Keywords:** fibronectin, glycoproteomics, N-glycans, integrin signals, cell migration

## Abstract

Directed cell migration is an important step in effective wound healing and requires the dynamic control of the formation of cell-extracellular matrix interactions. Plasma fibronectin is an extracellular matrix glycoprotein present in blood plasma that plays crucial roles in modulating cellular adhesion and migration and thereby helping to mediate all steps of wound healing. In order to seek safe sources of plasma fibronectin for its practical use in wound dressing, we isolated fibronectin from human (homo) and porcine plasma and demonstrated that both have a similar ability as a suitable substrate for the stimulation of cell adhesion and for directing cell migration. In addition, we also defined the N-glycosylation sites and N-glycans present on homo and porcine plasma fibronectin. These N-glycosylation modifications of the plasma fibronectin synergistically support the integrin-mediated signals to bring about mediating cellular adhesion and directed cell migration. This study not only determines the important function of N-glycans in both homo and porcine plasma fibronectin-mediated cell adhesion and directed cell migration, but also reveals the potential applications of porcine plasma fibronectin if it was applied as a material for clinical wound healing and tissue repair.

## INTRODUCTION

Wound healing is a dynamic process that consists of hemostasis, inflammation, proliferation and remodeling [1]. Fibronectin, an extracellular matrix (ECM) glycoprotein, plays important roles in the various different stages of wound healing, with its main function being cellular adhesion [2] and the mediation of cell migration [3]. Fibronectin interacts and activates cell surface integrin receptors that in turn recruit a series of cellular proteins involved in connecting with the actin cytoskeleton inside the cell; this initiates the formation of the integrin-based specialized adhesive organelles called focal adhesions (FAs) [4-8]. The coupling of actin cytoskeleton and ECM fibronectin via FAs dynamically drives directed cell migration during wound healing [4, 5, 9]. Initially, cell protrusions that are characterized by actin polymerization being to form a dense actin network; these are extended in the direction of migration, which is followed by attachment of the protrusions to the ECM fibronectin. This in turn forms nascent adhesions (new-born FAs). Subsequently, these nascent adhesions become mature and grow in size via myosin II-mediated contractile forces that are transduced along the bundles of actin filaments. Mature FAs transfer contractile forces from the actin cytoskeleton to the ECM fibronectin, thereby pulling the cell body forward. Finally, FA disassembly is accompanied by myosin II-driven contractile forces that retract the trailing edge of the cell from the ECM fibronectin [10-12]. The ECM fibronectin outside the cell that is linked to the actin cytoskeleton inside the cell via the FAs is thus closely associated during the process of wound healing with the dynamic control of cell adhesion and thence directed cell migration.

There are two forms of fibronectin, plasma fibronectin and cellular fibronectin. Plasma fibronectin is synthesized by hepatocytes and then released into the blood plasma [2, 13], while cellular fibronectin is produced by many cell types including fibroblasts, endothelial cells, myocytes and chondrocytes [14, 15]. During wound healing, it has been reported that plasma fibronectin accumulates to a remarkable extent in the *in vivo* wound after wounding occurs [16, 17]. This accumulation is crucial to the various functions of platelets, fibroblasts and endothelial cells and these include adhesion, migration and aggregation [2, 18]. The above indicates that plasma fibronectin is likely to serve as a suitable substrate for accelerating wound repair *in vivo*. Indeed, using animal models, the provision of matrix containing plasma fibronectin has been found to significantly improve epidermal cell adhesion and migration during the re-epithelialization process [19, 20]. Thus plasma fibronectin would seem to have clinical potential and should be able to help human wound healing and tissue repair. However, the application of plasma fibronectin to human wound healing has not been validated due to the lack of a reliable and inexpensive source of human plasma.

Given the importance of plasma fibronectin to wound healing, we set out to investigate whether plasma fibronectin isolated from pig (porcine plasma fibronectin) is able to play similar roles to plasma fibronectin isolated from humans (homo plasma fibronectin), namely the modulation of cell adhesion and directing of cell migration. We isolated fibronectin proteins from human and porcine plasma, and characterized the abilities of these two plasma fibronectins during cell attachment, cell adhesion and directed cell migration. In addition, we used glycoproteomic analysis to characterize the N-glycosylation sites and N-glycan structures present on homo and porcine plasma fibronectin. We found that the N-glycans present on plasma fibronectin have a role in the positive regulation of cell adhesion and in directing cell migration and that this occurs via the synergistic promotion of integrin-mediated adhesive signals.

## RESULTS

### Homo and porcine plasma fibronectin show comparable effects in terms of cell adhesion and migration

**Figure 1:**
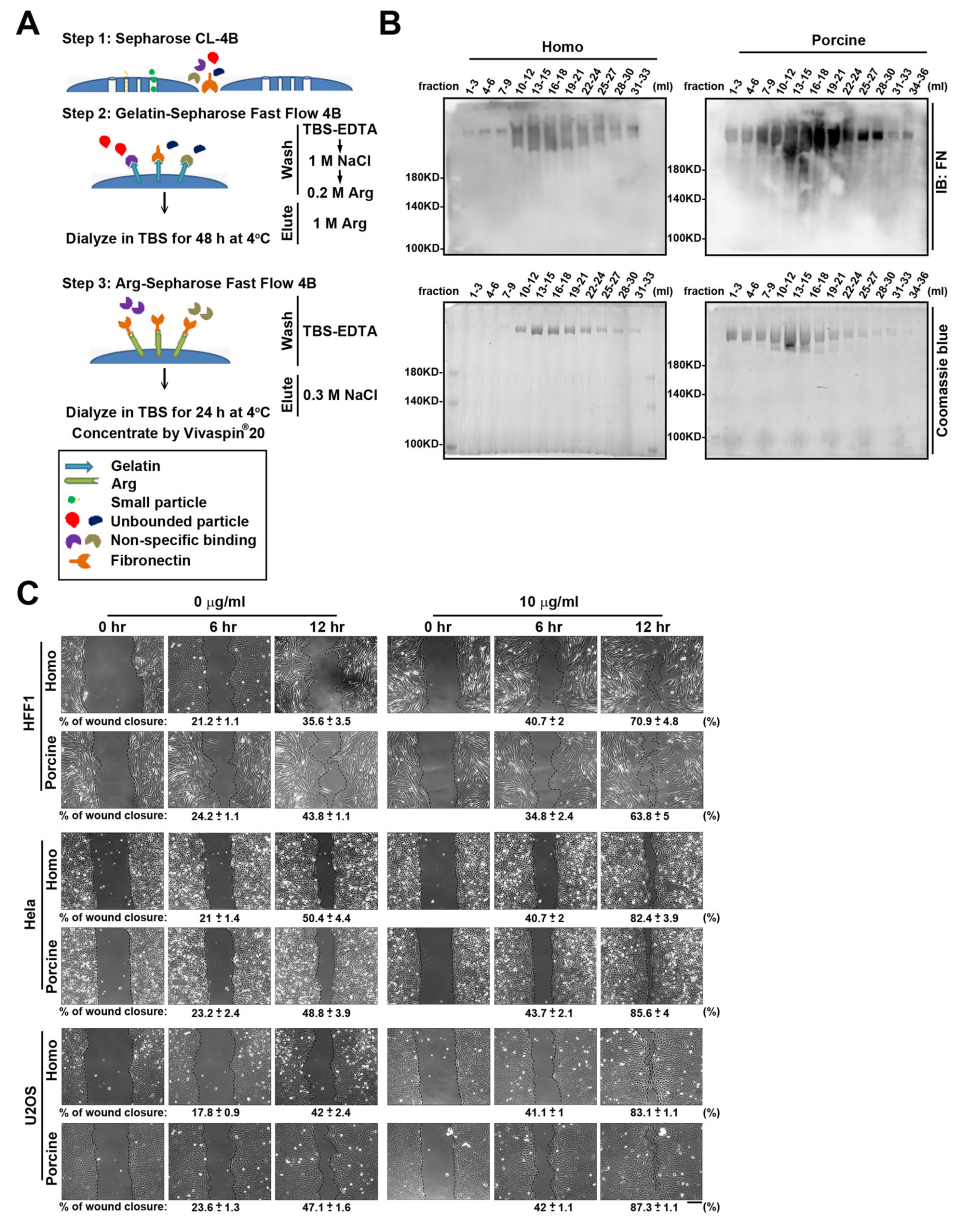
Fibronectin purification from homo and porcine plasma **(A)** Flow diagram of the purification steps used to isolate fibronectin from homo and porcine plasma. In step 1, cleared plasma was passed through a pre-column of Sepharose CL-4B in order to collect high molecular weight proteins. In step 2, the flow-through materials obtained from Sepharose CL-4B was loaded on a pre-column of gelatin-Sepharose Fast Flow 4B. After removing unbound proteins by sequentially washed with TBS-EDTA, 1 M NaCl and 0.2 M Arginine (Arg), the fibronectin was eluted with 1 M Arg and then dialyzed against TBS for 48 h at 4°C. In step 3, dialyzed material was applied to an Arg-Sepharose Fast Flow 4B column. After washing the column with TBS-EDTA, the fibronectin was eluted from the gel using 0.3 M NaCl/TBS-EDTA, and then dialyzed against TBS for 24 h at 4°C. Finally each of the fibronectins were concentrated using a Vivaspin 20 centrifugal concentrator (Molecular Weight Cut Off: 100 kDa). **(B)** The eluted fractions obtained from the Arg-Sepharose Fast Flow 4B column were analyzed by Western blotting using antibodies against fibronectin (FN) and Coomassie blue staining. **(C)** HFF1, Hela and U2OS cells plated on 6-well plates coated with 0 and 10 μg/ml homo or porcine plasma fibronectin for 16 h were assayed for wound-healing migration, which was monitored by time-lapse microscopy. The still images were obtained at the indicated times after wounding. The dotted lines mark the edge of the wound at the 0-h, 6-h and 12-h time points of wound-healing migration. Bar, 200 μm. Bottom: the percentage of wound closure was calculated using Metamorph software. Data are mean ± s.e.m. (n = 5 independent experiments).

Before demonstrating whether the N-glycans present on plasma fibronectin are involved in cell adhesion and migration, we first used a plasma fibronectin purification method to isolate high quality fibronectin proteins from homo and porcine plasma [21, 22] (Figure 1A). The two plasmas were passed individually through a pre-column of Sepharose CL-4B and the flow-through material that contained high molecular weight proteins was collected (Supplementary Figure 1A). These were subsequently loaded onto a column of gelatin-Sepharose Fast Flow 4B. The bound fibronectin attached to the column was eluted with 1 M Arg (Supplementary Figure 1B). The fractions containing each type of eluted fibronectin were pooled and dialyzed. Each dialyzed material was then individually re-applied to a column of Arg-Sepharose Fast Flow 4B. The specifically bound fibronectin was eluted with 0.3 M NaCl/TBS-EDTA (Figure 1B). These two pooled eluted fibronectin samples were then individually dialyzed and concentrated.

The protein sequences of homo and porcine fibronectin show high identity (94 %) (Supplementary Table 1 ; Ensembl database), which implies that they should have similar abilities when promoting cell adhesion and migration. To compare the functionalities of the isolated fibronectin from homo and porcine plasma, we carried out wound-healing migration assays using cells that had been plated on the fibronectin-coated plates. This revealed that the isolated homo and porcine fibronectin proteins exhibited similar wound closure effect using U2OS, HFF1 and Hela cells (Figure 1C). Therefore, homo and porcine fibronectins would seem to possess comparable capabilities in terms of regulating cell migration.

**Figure 2:**
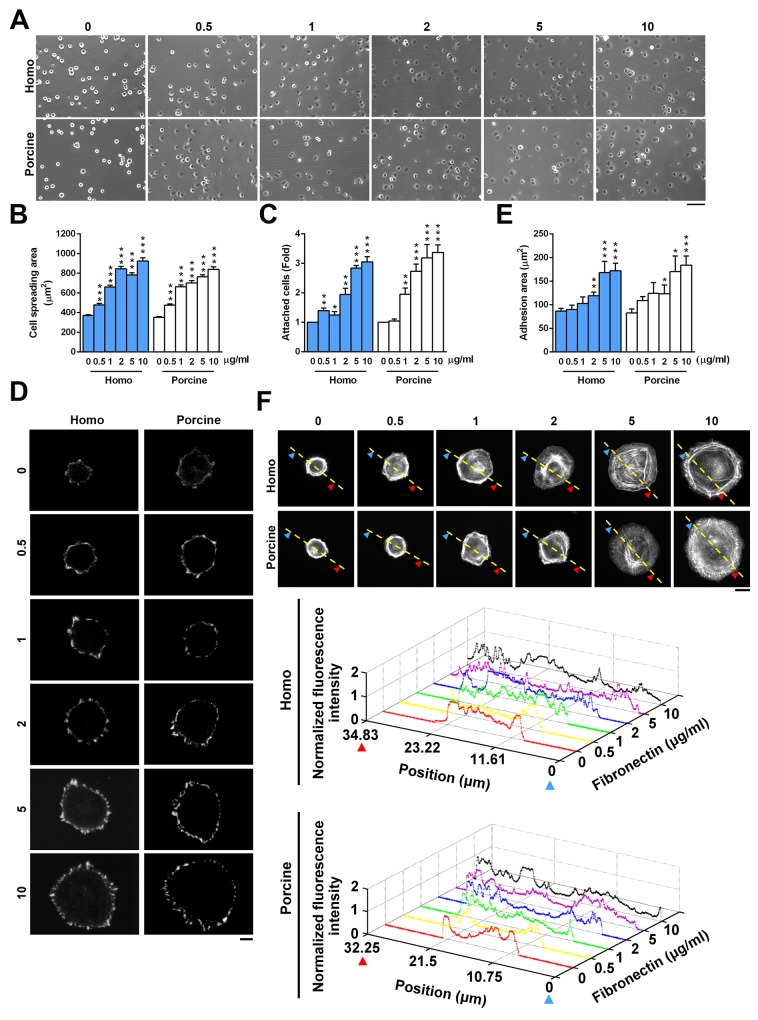
Cell adhesion in response to homo and porcine plasma fibronectin **(A)** U2OS cells were plated on coverslips coated with the indicated fibronectin concentration (μg/ml) for 30 min and then the images were taken by phase contrast microscopy. Bar, 100 μm. **(B)** The plot shows the area of cells spreading on coverslips coated with the indicated fibronectin concentration (μg/ml). Data are mean ± s.e.m. (n = 80 cells in every condition). ***p < 0.001. **(C)** U2OS cells were plated on the indicated concentration of fibronectin for 30 min and then their cell attachment was measured. Fold of cells remaining attached on the indicated concentration of fibronectin relative to that on 0 μg/ml fibronectin. Data are mean ± s.e.m. (n = 8 independent experiments). *p < 0.05; **p<0.01; ***p < 0.001. **(D)** TIRFM images of U2OS cells that had been plated for 1.5 h on coverslips coated with the indicated fibronectin concentration and immunostained with paxillin. Bar, 10 μm. **(E)** Plot shows the sum of the total area of paxillin-marked focal adhesions within a cell versus the fibronectin concentration. Data are mean ± s.e.m. [homo: n =15 cells (0 μg/ml), 20 cells (0.5 μg/ml), 13 cells (1 μg/ml), 20 cells (2 μg/ml), 18 cells (5 μg/ml), 15 cells (10 μg/ml); porcine: n= 9 cells (0 μg/ml), 14 cells (0.5 μg/ml), 11 cells (1 μg/ml), 9 cells (2 μg/ml), 11 cells (5 μg/ml), 10 cells (10 μg/ml)]. *p < 0.05; **p<0.01; ***p < 0.001. **(F)** Confocal images of U2OS cells that were plated for 1.5 h on coverslips coated with the indicated fibronectin concentration (μg/ml). Bar, 10 μm. (Bottom) Relative fluorescence intensity taken along the line highlighted in the confocal image with the edge being marked with arrows and the distance.

To determine whether homo and porcine fibronectins are comparable when regulating adhesion strength and the organization of F-actin, we initially compared the effect of homo and porcine fibronectin on cell spreading and adhesion. The area of cell spreading was measured after 30 min using U2OS cells that had been seeded onto plates coated with increasing concentrations of homo or porcine fibronectin (Figure 2A). The results revealed that the area of cell spreading increased as the concentration of fibronectin increased for both fibronectins (Figure 2B). Cell adhesive capacity was also quantified and this revealed that increasing concentrations of homo or porcine fibronectin promoted cell adherence to fibronectin (Figure 2C). Next, we immunolabelled and visualized the cellular pattern of the FA marker paxillin (Figure 2D) using cells seeded on increasing concentration of homo or porcine fibronectin for 1.5 h. The results showed an increased area of adhesion (μm^2^) as the concentration of each fibronectin increased; the quantification was in terms of the area of paxillin-marked adhesion (Figure 2E). To further determine whether there were fibronectin-dependent changes in adhesion strength that affected actin cytoskeleton organization, U2OS cells were plated onto plates coated with increasing concentration of homo or porcine fibronectin for 1.5 h; these were then immunolabelled for F-actin. The results revealed a dense network of F-actin covering the peripheral region of cells for both fibronectins, and the organization of the actin cytoskeleton spread to entire cells as the size of the cells increased (Figure 2F). Similar results were observed using Hela cells (Supplementary Figure 2) and human foreskin fibroblasts (HFF1) cells (Supplementary Figure 3). The two isolated fibronectins have been shown to be able to geometrically patterned and used as substrates to control the microenvironment of individual mesenchymal stem cells; this in turn modulated the decision of cells to adhere and spread [23-25]. We found that patterned substrates coated with increasing concentrations of either homo or porcine fibronectin had similar effects in terms of controlling cell shape, modifying the degree of cell spreading on micro-patterned areas and affecting the organization of paxillin-marked focal adhesions and F-actin (Supplementary Figure 4). Thus it would seem that homo and porcine fibronectins are equally capable of promoting cell adhesion strength, modifying actin cytoskeleton organization and affecting cell migration and that these changes occur in a fibronectin concentration-dependent manner.

### Characterization of the N-glycosylation sites present on homo and porcine fibronectin

Fibronectin, a large glycoprotein, is one of the best characterized cell adhesion-promoting ECM proteins. Although the cell-binding domain of fibronectin has been well-explored [26], the role of attached glycans on the protein’s binding functions remains unclear. In order to define the N-glycosylation sites and N-glycan structures present on homo and porcine fibronectin, each isolated fibronectin was analyzed by LC-MS/MS-based glycopeptide sequencing and the peptides identification using the Orbitrap Fusion Tribrid MS system. The isolated fibronectin proteins were reduced, alkylated, trypsin digested and separated by liquid chromatography before the Orbitrap survey MS (MS^1^) scan, which was followed by a decision step for the data dependent acquisition of higher collision energy dissociation (HCD)-MS^2^. Tandem mass spectra were generated and directly searched against a sample dependent fibronectin protein database, using the Byonic™ search engine and its default built-in N-glycan library. The glycosyl composition of the N-glycans associated with their respective carrier peptide backbones were thus identified and likely structures deduced [27]. As an example, a precursor glycopeptide (*m/z* 1035.6478) from homo fibronectin with a charge state of 4^+^ was identified by the Byonic™ software [27] as HEEGHMLNCTCFGQGR glycosylated at the N542 site with an N-glycan having a composition HexNAc(4)Hex(5)NeuAc(2). This was based on the peptide backbone ion carrying one (Y1 ion) to several glycosyl residues, along with several peptide cleavage b and y ions (Supplementary Figure 5).

**Figure 3:**
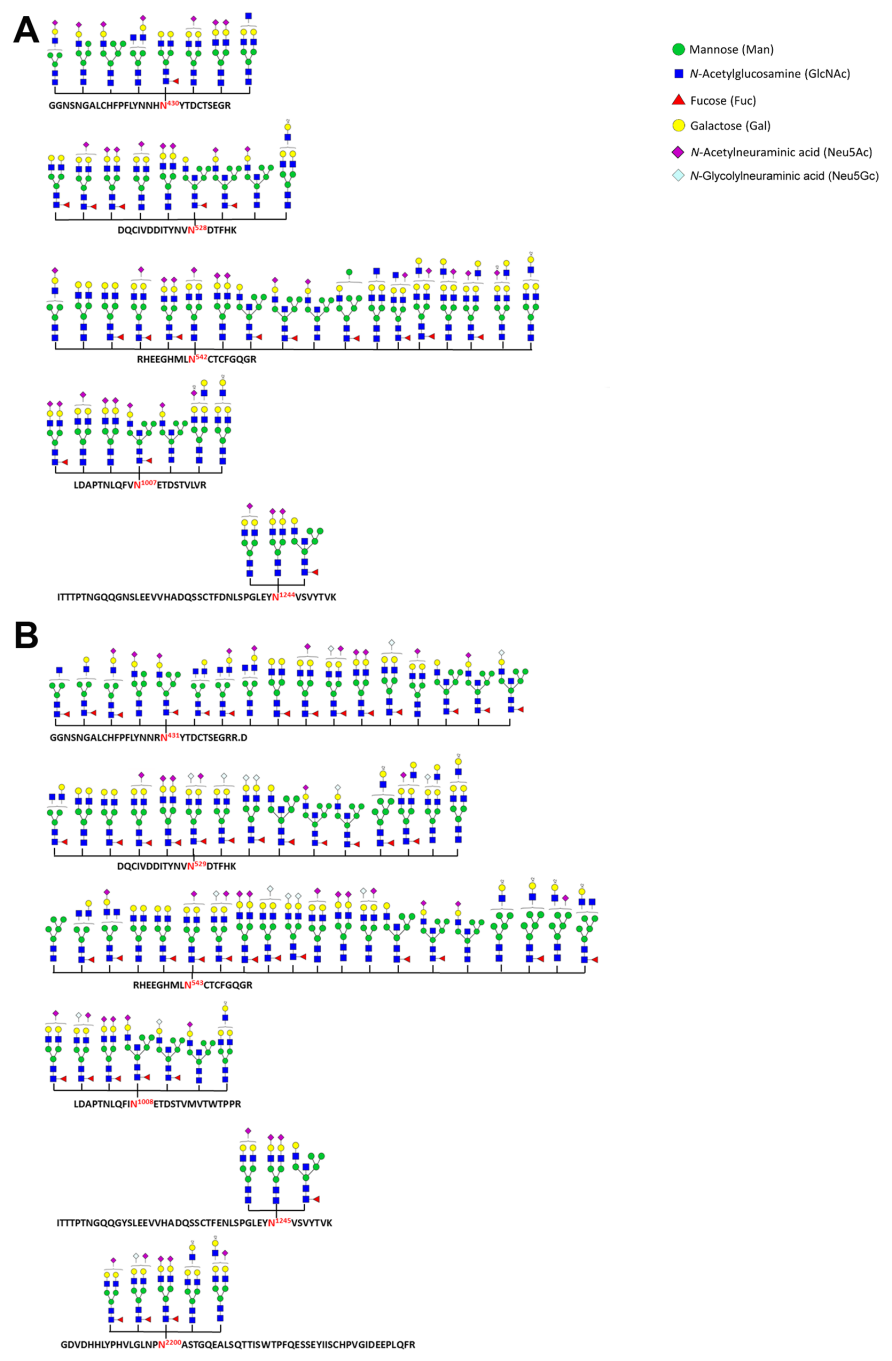
N-glycosylation sites and N-glycans identified as present within homo and porcine plasma fibronectin A database search using Byonic™ identified N-glycan + peptide combinations from homo **(A)** and porcine **(B)** plasma fibronectin with each site being shown using the standard symbol nomenclature for glycans system [48]. The N-glycan structures are mostly inferred from the assigned glycosyl compositions (see Supplementary Tables 2 and 3) without any further attempt to verify their isomeric arrangement.

In total, five N-glycosylation sites (N430, N528, N542, N1007 and N1244) were identified in the homo fibronectin (Figure 3A), while six novel sites (N431, N529, N543, N1008, N1245 and N2200) were identified in the porcine fibronectin (Figure 3B). Interestingly, all the N-glycans of the homo and porcine plasma fibronectin that were detected are either hybrid or complex-type N-glycans without any significant level of high mannose structures; the results are summarized in Supplementary Tables 2 and 3. A majority of the deduced N-glycans in both fibronectin samples are sialylated and/or fucosylated (Figure 3). As expected, both forms of sialic acid, namely N-acetylneuraminic acid (Neu5Ac) residues and N-glycolylneuraminic acid (Neu5Gc) residues, were found in the porcine plasma fibronectin. However, only the Neu5Ac residue was identified in the homo plasma fibronectin (Figure 3). It is not surprising that there are no Neu5Gc residues associated with the homo plasma fibronectin because the human gene, CMP-N-acetylneuraminic acid hydroxylase (CMAH), which synthesizes Neu5Gc, is irreversibly mutated in humans. Thus, while Neu5Gc is present in most mammals, it is not present in humans [28-31]. Taking the above findings as a whole it can be concluded that the differences between homo and porcine plasma fibronectin in terms of site-specific N-glycans are relatively minor.

### The controlling of cell adhesion by the N-glycans present on plasma fibronectin

**Figure 4:**
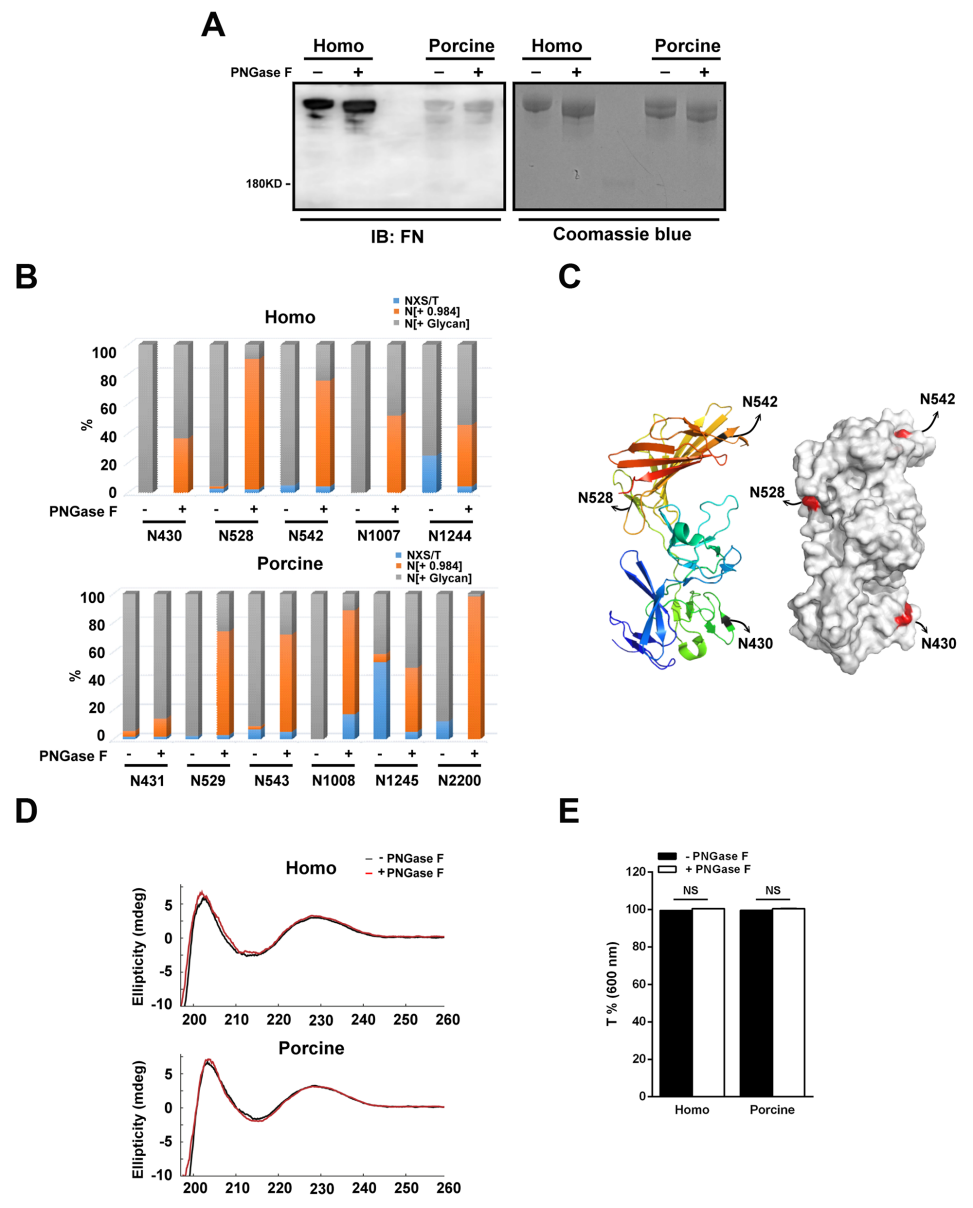
N-glycans present within human and porcine fibronectin are digested with PNGase F **(A)** The homo and porcine plasma fibronectin treated with (+) or without (-) PNGase F were analyzed by Coomassie blue staining and Western blotting using antibodies against fibronectin (FN). **(B)** Homo and porcine fibronectin with (+) or without (-) PNGase F digestion were analyzed using the spectral counts of the non-N-glycosylated (NXS/T), N-glycosylated (N[+ Glycan]) and de-N-glycosylated (N[+ 0.984]) residues at each individually detected N-glycosylation site. A plot of the spectral counts of NXS/T, N[+ Glycan] and N[+0.984] was calculated as a percentage to total spectral counts of all detected N-glycosylation sites and this demonstrated efficient de-glycosylation at certain N-glycosylation sites. **(C)** A ribbon model (left) and surface contour image (right) were obtained from the Protein Data Bank using access code 3M7P (homo fibronectin amino acid 297-604). The N-glycosylation sites (N430, N528 and N542) are highlighted. **(D)** Comparison of the CD curves of homo and porcine fibronectin before (black) and after (red) PNGase F digestion. **(E)** Plot shows the optical transmittance rates at 600 nm of homo and porcine fibronectin, with (+) and without (-) PNGase F digestion. Data are mean ± s.e.m. (n = 3 independent experiments). NS, no significance.

To understand the role of N-glycans in relation to the cell-binding functions of plasma fibronectin, we first used peptide-N-glycosidase F (PNGase F) to cleave the N-linked glycans from their asparagine residues within the fibronectin protein. Coomassie blue staining and immunoblotting of the isolated plasma fibronectin revealed that the PNGase F-treated fibronectin migrated more rapidly than untreated fibronectin (Figure 4A). The polyclonal antibodies against fibronectin that we used in the present study were generated using the C-terminus of homo fibronectin (amino acids 2087-2386), which has a 91% amino acid identity to the same region of porcine fibronectin. Although still quite high, the lack of identity may explain the lower level of staining obtained with the porcine fibronectin. There is probably being weaker binding specificity of the fibronectin antibodies to the porcine fibronectin.

In order to confirm the efficiency of de-glycosylation by PNGase F, LC-MS/MS-based glycopeptide analysis was performed to evaluate the relative changes in the amount of de-N-glycosylated residues across the detected N-glycosylation sites between the untreated and PNGase F-treated plasma fibronectins. Spectral counts (the number of detected peptides) are able to represent the relative changes in protein abundance when similarly prepared samples are used [32], and therefore we calculated the ratio of the spectral counts of the non-N-glycosylated (NXS/T), the N-glycosylated (N[+ Glycan]) and the de-N-glycosylated (N[+ 0.984]) residues relative to the total spectral counts for the individual detected N-glycosylation sites; these are presented as a percentage. When the percentage of de-N-glycosylated residues across the detected N-glycosylation sites was compared between the untreated and PNGase F-treated plasma fibronectin, it was revealed that the PNGase F is able to access all detected N-glycosylation sites on both the homo and porcine plasma fibronectins (Figure 4B). However, the treatment with PNGase F was not 100% efficient when de-glycosylation was carried out on plasma fibronectin in its native form. This can be seen in the results shown in Figure 4A where the PNGase F-treated fibronectins still display higher molecular weights than predicted size (220 kDa). Of the identified N-glycosylation sites, we found that PNGase F efficiently removed the N-glycans (> 60%) of homo fibronectin at N528 and N542 but not N430 (Figure 4B), although all three sites are highly solvent accessible (Figure 4C). It is not known whether two of the N-glycosylation sites, N1077 and N1244, are on the surface of the fibronectin protein because there is no reported structure. In addition, we found that the N-glycans at N529, N543, N1008 and N2200 of porcine fibronectin were efficiently removed (> 60%) (Figure 4B). These findings suggest that PNGase F is able to access all the identified N-glycosylation sites and is able to remove the glycan efficiently at certain N-glycosylation sites.

Cleavage with PNGase F converts each asparagine residue into aspartic acid residue and therefore we applied circular dichroism (CD) spectroscopy to verify whether there were changes to the structure of fibronectin after PNGase F treatment. The CD curve of untreated plasma fibronectin (Figure 4D) was found to be similar to that reported previously [21]. When untreated and PNGase F-treated plasma fibronectin were compared, there was no significant difference in the CD spectrum (Figure 4D), indicating that the secondary structure components of these different forms of fibronectin largely similar. In addition, we have also ascertained that neither precipitation nor aggregation occurred after PNGase F digestion by turbidity testing [33] (Figure 4E). The optical transmittance rates at 600 nm of the two untreated fibronectins are both about 100% (homo: 99.4%; porcine: 99.6%), while those of both PNGase F-treated fibronectins are also about 100% (homo: 100%; porcine: 100%) (Figure 4E). Therefore, it would seem that the structural integrity and physical properties, including solubility, of the two fibronectins are not influenced by partial removal of N-glycans.

**Figure 5:**
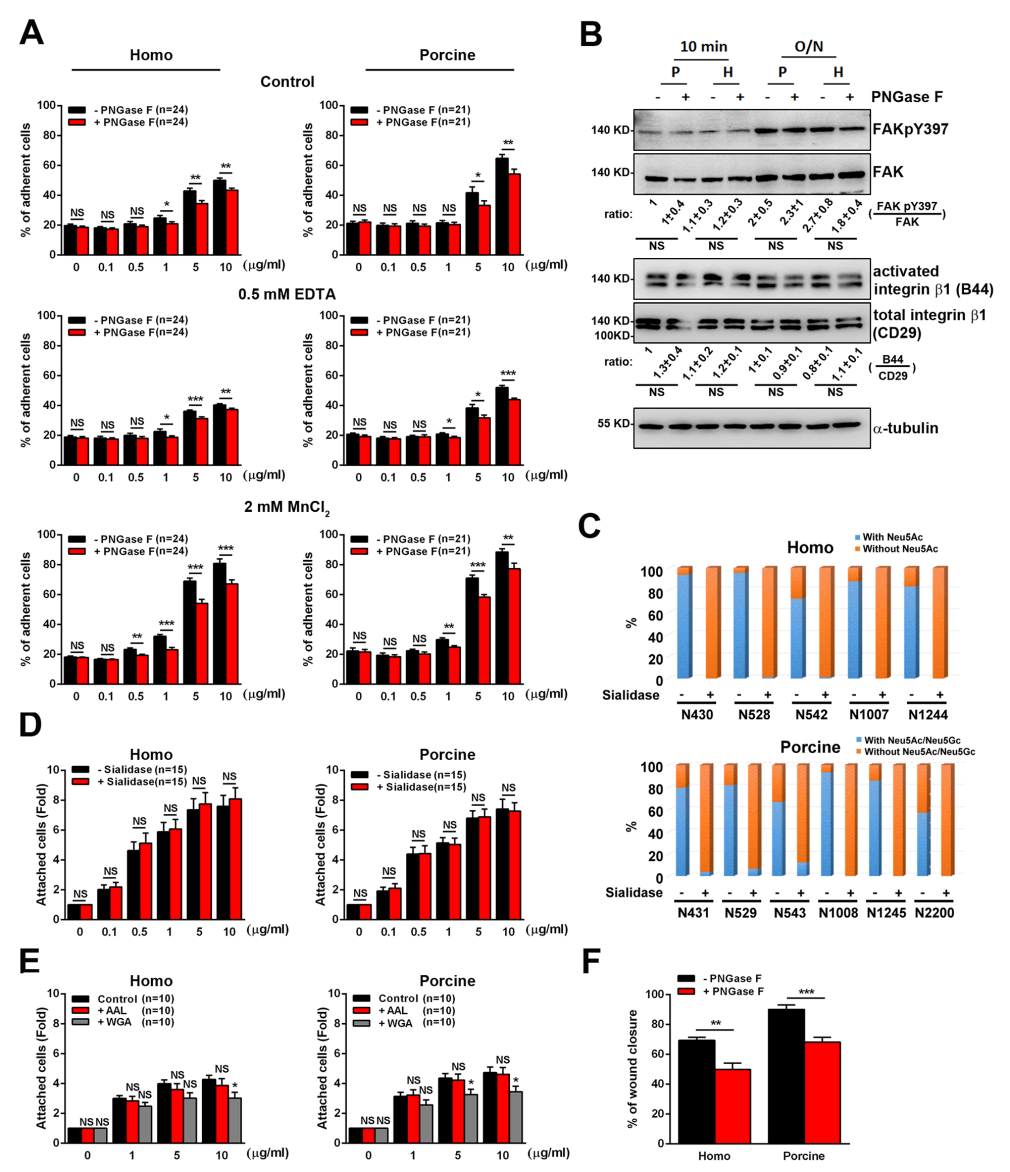
Cell adhesion and migration in response to the N-glycosylation of human and porcine fibronectin **(A)** Cells were pre-incubated with serum-free medium containing H_2_O (control), serum-free medium containing 0.5 mM EDTA, or culture medium containing 2 mM MnCl_2_ before plating on the indicated concentrations of fibronectin [FN] treated with (+) or without (-) PNGase F. Cells were plated on [FN] for 10 min and then their attachment was measured. Percentage of cells remaining attached on [FN] was shown. Data are the mean ± s.e.m. (n = number of independent experiments). *p < 0.05; **p<0.01; ***p < 0.001; NS, no significance. **(B)** Cell lysates from U2OS cells plated on 10 μg/ml FN treated with (+) or without (-) PNGase F for 10 min or 16h (overnight) were analyzed by Western blotting using antibodies against FAK, FAKpY397, total integrin β1 (CD29), activated integrin β1 (B44) and α-tubulin. Bottom: ratio indicates the expression of FAKpY397 normalized against FAK and activated integrin β1 (B44) against to total integrin β1 (CD29) from Western blotting. The ratio in the indicated conditions is calculated relative to that in the first lane in the same gel. Data are the mean ± s.e.m. (n = 5 independent experiments). NS, no significance. **(C)** Homo and porcine plasma fibronectin with (+) or without (-) α2-3,6,8 Neuraminidase (Sialidase) digestion were analyzed to assess the spectral counts of N-glycosylated residues with or without sialic acids at the various individually detected N-glycosylation sites. The plot shows the percentage of the spectral counts of the N-glycosylated residues with or without sialic acids relative to the total spectral counts for all detected N-glycosylation sites. The results demonstrated an efficient process for the trimming of sialic acids across the various N-glycosylation sites. **(D)** Cells in serum-free medium were plated for 20 min on [FN], treated with (+) or without (-) α2-3,6,8 Neuraminidase (Sialidase). The fold of cells remaining attached on [FN] relative to that on 0 μg/ml fibronectin is shown. Data are the mean ± s.e.m. (n = number of independent experiments). NS, no significance. **(E)** Cells in serum-free medium were seeded on plates coated with [FN], accompanied by ddH_2_O, AAL or WGA for 20 min. The fold of cells remaining attached on [FN] relative to that on 0 μg/ml fibronectin was measured. Data are the mean ± s.e.m. (n = number of independent experiments). *p < 0.05; NS, no significance. **(F)** Cells were plated on homo or porcine fibronectin, treated with (+) or without (-) PNGase F and then assayed for wound-healing migration. The percentage of wound closure at 10-h time point of wound-healing migration was calculated using Metamorph software and plotted. Data are the mean ± s.e.m. [homo: n = 5 (-PNGase F), n = 6 (+PNGase F) independent experiments; porcine: n = 6 (-PNGase F), n = 5 (+PNGase F) independent experiments]. **p<0.01; ***p < 0.001.

Next we wish to explore how N-glycosylation affects cell adhesions. To this end we measured the adhesive ability of cells by allowing the cells to attach to untreated and PNGase F-treated fibronectin. We found that the adhesion of U2OS cells was lower when cells were plated on increasing concentration of homo or porcine fibronectin treated with PNGase F, compared to being plated on increasing concentrations of untreated fibronectin (Figure 5A). In detail, with the addition of 10ug/ml of homo and porcine fibronectin upon PNGase F treatment, adhesion was significantly reduced to about 7% and 10%, respectively. This suggests that N-glycans on plasma fibronectin is involved in cell adhesion via an ability to strengthen the binding affinity of fibronectin to the cell surface. Based on the results in Figure 4B, we speculate that the N-glycosylation sites that can be efficiently de-glycosylated by PNGase F (homo: N528 and N542; porcine: N529, N543, N1008 and N2200) are likely to play an important role in the adhesion function of fibronectin.

To gain further insight into what is the possible mechanism behind the adhesive role of N-glycans with respect to plasma fibronectin, we investigated whether the present of N-glycans significantly cooperates with integrin-mediated signals for cell adhesion. If N-glycans do support cell adhesion independently and cooperate with integrin-mediated signals to promote cell adhesion, the treatment of cells with EDTA, which blocks integrins-mediated cell adhesion by chelating calcium [34] and suppress adhesion on 10ug/ml homo and porcine fibronectin to about 10% and 12%, respectively (Figure 5A), should eliminate cell adhesion to PNGase F-treated fibronectin significantly, compared to untreated fibronectin. Indeed, when U2OS cells were plated on increasing concentration of PNGase F-treated fibronectin in the presence of EDTA, the cell-fibronectin association was suppressed significantly (Figure 5A). In addition, treatment of these U2OS cells with MnCl_2_, which induces a conformational change in the integrin receptors in favor of a high-affinity state [35] and increase adhesion on 10ug/ml homo and porcine fibronectin to about 30% and 24%, respectively, did not limit the ability of N-glycans to support cell-fibronectin association (Figure 5A). Furthermore, the integrin signals (activated integrin β1 and FAKpY397) of U2OS cells was not suppressed when cells were plated on homo or porcine fibronectin treated with PNGase F, compared to being plated on untreated fibronectin (Figure 5B). These findings indicate that the adhesion enhancement effect of N-linked glycans with respect to plasma fibronectin involves a mechanism that revolves around various cell surface molecules, such as glycoconjugates or lectins, and involves cooperative integrin-fibronectin engagement; this then leads to the modulation of cell adhesion.

Based on the results of the site-specific N-glycosylation profiling (Figure 3), each of the N-glycosylation sites carries sialylated and/or fucosylated glycans. To further determine the key glycans responsible for the functioning of fibronectin during adhesion enhancement, we first used α2-3,6,8 Neuraminidase (sialidase) to cleave sialic acids from fibronectin (Figure 5C). We also used lectins (Aleuria aurantia agglutinin (AAL) or wheat germ agglutinin (WGA)) to block the fucose or N-acetylglucosamine present on fibronectin molecules [36]. We found that both the removal of sialic acids from fibronectin (Figure 5D) or the blocking of fucose on fibronectin molecules using AAL (Figure 5E) did not affect the cell-fibronectin association, while, on the other hand, N-acetylglucosamine does have a significant effect on cell adhesion (Figure 5E). These findings suggest that N-acetylglucosamine is the key glycan molecule present on fibronectin involved in supporting cell adhesion.

Next we focused on the relationship between plasma fibronectin N-glycosylation and cell migration. U2OS cells were seeded on plates coated with untreated or PNGase F-treated plasma fibronectin and subjected to wound-healing migration assays. When we analyzed the percentage of wound closure over a 10-h migration period, it was found that, compared with cells seeded on untreated fibronectin-coated plates, cells cultured on the plates coated with PNGase F-treated fibronectin displayed a much lower percentage of wound closure (Figure 5F). These findings reveal the importance of the N-linked glycans present on both homo and porcine plasma fibronectin to the control of cell-fibronectin attachment, cell adhesion and cell migration capability. Taken together, our findings suggest that the N-glycans present on plasma fibronectin is involved in mediating the cell-fibronectin association and in cooperative integrin-mediated adhesion signaling. These in turn control cell adhesion; the process consequently enhances cell migration.

## DISCUSSION

**Figure 6:**
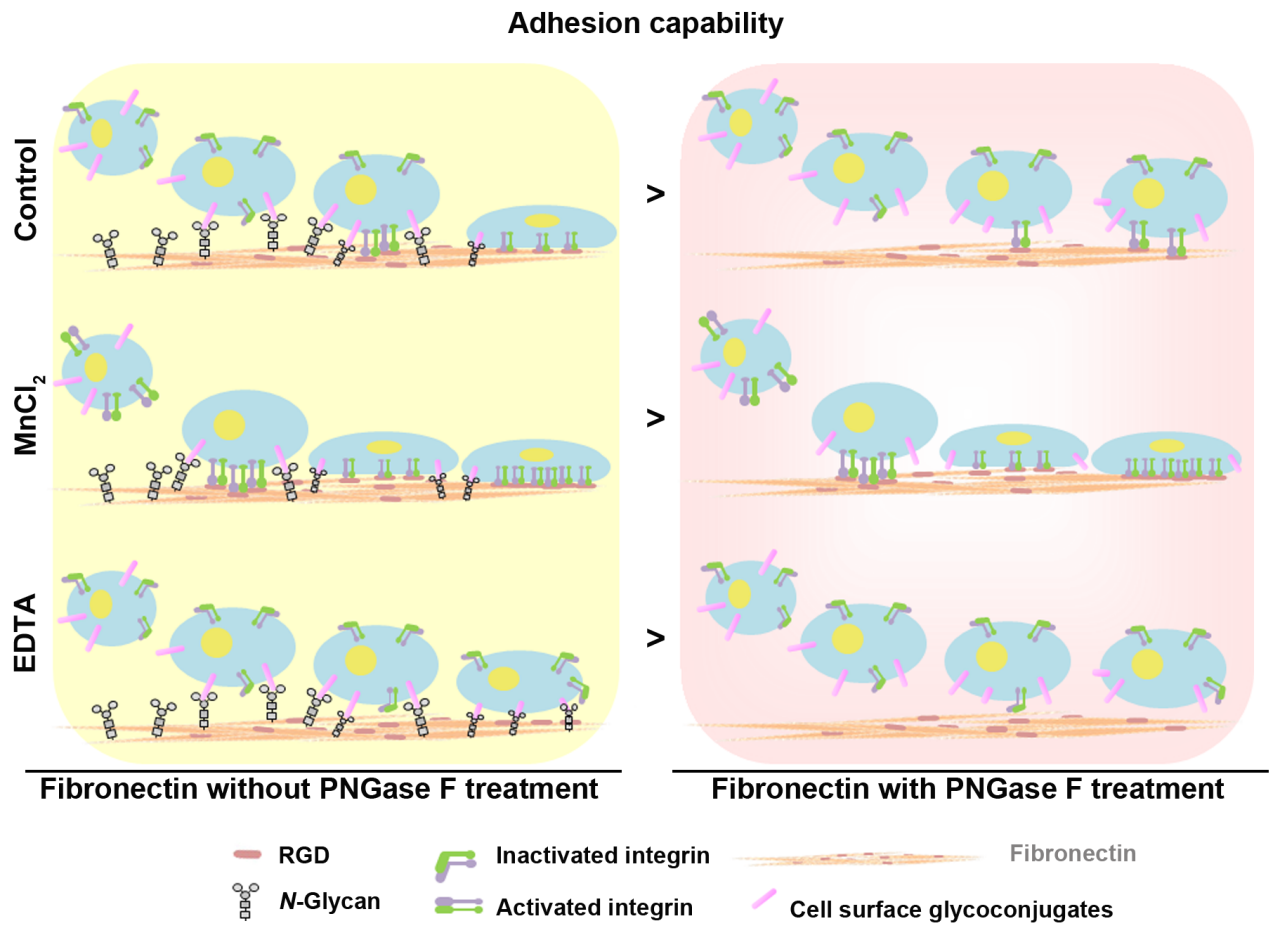
Model showing the synergistic effects of N-glycans and the RGD sequence of fibronectin on the regulation of cell adhesion capability N-glycans act synergistically with the integrin-binding regions (RGD sequence) of plasma fibronectin to support cell adhesion and wound-healing migration.

Our study initially examined the effect of porcine and homo plasma fibronectin on cell adhesion and wound-healing migration and the findings revealed that porcine plasma fibronectin has potential as a wound dressing material and is likely to useful in clinical wound healing and tissue repair. Importantly, we also profiled the N-glycosylation sites present in and N-glycans present on homo and porcine plasma fibronectin and pinpointed a novel role for N-glycosylation regarding the regulation of cell attachment, adhesion and migration capability. To further understand the role of N-glycans, we examined whether cell attachment to plasma fibronectin occurred in a N-glycan-dependent manner. We focused on N-glycan-dependent cell adhesion to plasma fibronectin and showed that, without integrin activation (EDTA treatment), the attachment of N-glycans to cell surfaces is the only support for cell adhesion, while integrin activation (MnCl_2_ treatment) [35] is not able to mask the effect of N-glycan-mediated cell-fibronectin attachment (Figure 6). Thus we have demonstrated that there is synergistic signaling between N-glycans and the integrin-binding regions of plasma fibronectin during cell adhesion and wound-healing migration.

Fibronectin is a large glycoprotein and homo fibronectin contains seven potential N-glycosylation sites (N430, N528, N542, N877, N1007, N1244 and N2108) [37-41]. However, our results were able to only identify five N-glycosylation sites (N430, N528, N542, N1007 and N1244) in homo plasma fibronectin (Figure 3A). Although 90.11% sequence coverage of homo plasma fibronectin was achieved in the LC-MS/MS-based glycopeptide analysis, glycopeptides at N877 and N2108 were not detected in the peptide mixtures. One possible reason in that digestion with trypsin has resulted in either very short or very long peptides that were unable to be deteceted in our system. The five identified N-glycosylation sites contain major complex-type oligosaccharides and few hybrid-type oligosaccharides that are partially sialylated and fucosylated (Figure 3A and Supplementary Table 2). The process of post-translational modification depends on the specific enzymes and therefore the level of modification of a fibronectin molecule across individuals may differ. For example, core fucosylation is catalyzed by α1,6-fucosyltransferase (Fut8), expression level of which is regulated and this enzyme is also important for liver regeneration [42]; these factors may result in different level of fucosylation across individuals. This raises the possibility that the amount of glycans on plasma fibronectin could differ from blood donor to blood donors. Notwithstanding this possibility of individual variation, this study is the first analysis of N-glycan composition of plasma fibronectin. In addition, our results have revealed the presence of six novel N-glycosyaltion sites (N431, N529, N543, N1008, N1245 and N2200) on porcine plasma fibronectin that involve fucose, Neu5Ac and Neu5Gc. The glycopeptide involving the N-glycosylation site N2200 was detected only in porcine fibronectin, but not in homo fibronectin. Our study is significant because it has identified the site-specific N-glycosylation in these two plasma fibronectins and has revealed similar N-glycan patterns within the plasma fibronectins from humans and pigs. However, it is possible that the clinical application of porcine fibronectin may cause an aberrant immune response due to the presence of Neu5Gc, because of the absence of endogenous Neu5Gc in humans [28-31]. Since the trimming sialic acids from plasma fibronectin did not change the proteins ability regarding cell adhesion (Figure 5C and 5D), the removal of the sialic acids from porcine plasma fibronectin might be useful when included in the production process for novel wound dressing materials.

Our results suggest a model in which N-glycans regulate cell adhesive capability by modulating cell-fibronectin interactions, which in turn leads to the integrin-mediated signals that lead to cell migration. The Arg-Gly-Asp (RGD) region, not the identified N-glycosylation sites (N430, N528, N542, N1007 and N1244), of homo plasma fibronectin are found within the cell-attachment region (aa.1267-1540). This supports the notion that the RGD region of fibronectin mainly mediates the engagement and activation of the cell surface integrin receptors [43]. N430, N528 and N542 are within the collagen-binding region (aa. 308-608), while N1007 and N1244 are within type-III domain between the collagen-binding (aa. 308-608) and cell-attachment region (aa.1267-1540). These findings suggest that unknown cell surface molecules, such as glycoconjugates or lectin, may be involved in recognizing the site-specific N-glycans that help to enhance cell-fibronectin attachment. Our findings also reveal that removal of these site-specific N-glycans suppresses cell attachment and, in accordance with this finding, integrin receptor inactivation (EDTA treatment) causes a significant suppression of cell-fibronection adhesion (Figure 5A). Importantly, N-glycan-cleaved plasma fibronectin is able to significantly suppress wound-healing migration (Figure 5F). This suggests a hypothesis in which the N-glycans in plasma fibronectin are coupled to cell surface molecules such as glycoconjugates or lectins. Such a process will minimize the distance between the RGD region and cell surface integrin receptors allowing the propagation of cell adhesion that then drives cell migration.

Our findings have revealed a previously unrecognized role for N-glycans in plasma fibronectin, namely that this protein is responsible for cell adhesion and cell migration (Figure 5). However, whether the O-glycans present as part of plasma fibronectin mediate the cell-fibronectin association remains an open question, because no enzymatic O-glycan removal method is available for native fibronectin. It is known that O-glycans, as well as N-glycans, involve sialic acids, fucose, N-acetylglucosamine, galactose and other moieties. In this context sialidases, fucosidases or lectins are able to remove or block sugar residues present in both O-glycans and N-glycans. We found that trimming sialic acids or blocking fucose on plasma fibronectin did not result in a restriction in the adhesive ability of the attached cells (Figure 5D and 5E), in spite of the fact that cell surface oligosaccharides, such as sialic acid [44] and fucose [45], are able to enhance cell adhesion. Our findings have also revealed the important role of N-acetylglucosamine relation to fibronectin regarding support for cell adhesion (Figure 5E). Nevertheless, we do not know whether the adhesion function is mediated by N-acetylglucosamine-linked O-glycans or by N-acetylglucosamine-linked N-glycans or by both. In addition, how the oligosaccharides in plasma fibronectin engage with cell surface molecules, such as glycoconjugates or lectins and bring about the specific signals associated with cell adhesion also remains to be determined.

## MATERIALS AND METHODS

### Cells and plasma

U2OS (human bone osteosarcoma cell line) and Hela (human cervical adenocarcinoma epithelial cell line) were gifts from Prof. R.-H. Chen’s laboratory (Academia Sinica, Taipei, Taiwan) and were maintained in DMEM-high glucose (Invitrogen) supplemented with 10% FBS (Invitrogen) and 1% antibiotic solution (penicillin and streptomycin; Invitrogen) under 5% CO_2_. HFF1 (human foreskin fibroblasts) cells were purchased from ATCC and were maintained in DMEM-high glucose supplemented with 15% FBS and 1% antibiotic solution (penicillin and streptomycin) under 5% CO_2_. Human mesenchymal stem cells (MSCs) were purchased from Lonza and were maintained in DMEM-low glucose supplemented with 10% FBS (Hyclone) and 1% antibiotic solution (penicillin and streptomycin) under 5% CO_2_.

The homo plasma was obtained from human blood donated by blood donors. All methods related to human blood were carried out in accordance with relevant guidelines and regulations. All experiments protocols related to human blood were approved by the Ethics Committee of the Institutional Review Board (IRB) of National Yang-Ming University. Informed consent was obtained from all subjects. The porcine plasma was obtained from CHAISHAN FOODS CO., LTD.

### Antibodies

The sources of the antibodies and their dilutions were as follows: mouse anti-paxillin (BD Bioscience 610052; dilution for immunofluorescence: 1/1000); rabbit anti-fibronectin (Santa Cruz Sc-9068; dilution for Western blotting: 1/500); rabbit anti-FAK (invitrogen AHO0502; dilution for Western blotting: 1/100); rabbit anti-FAK-pY397 (GeneTex GTX129840; dilution for Western blotting: 1/500); rabbit anti-integrin · 1 (CD29) (GeneTex GTX128839; dilution for Western blotting: 1/1000); mouse anti-integrin β1 (B44) (Millipore MAB2259Z; dilution for Western blotting: 1/100); mouse anti-α-tubulin (Sigma T1568; dilution for Western blotting: 1/4000); Alexa Fluor 488 phalloidin (Invitrogen A12379; dilution for immunofluorescence: 1/300); Alexa Fluor 568-anti-mouse IgG (Invitrogen A11031; dilution for immunofluorescence: 1/300); HRP-AffiniPure goat anti-mouse IgG (Jackson ImmunoResearch 115-035-174; dilution for immunofluorescence: 1/50000); and HRP-AffiniPure mouse anti-rabbit IgG (Jackson ImmunoResearch 211-032-171; dilution for immunofluorescence: 1/50000).

### The plasma fibronectin purification procedure

The plasma fibronectins were purified from homo plasma (donated blood) and porcine plasma (CHAISHAN FOODS CO., LTD.) using immobilized gelatin and Arg affinity chromatography as described previously (Figure 1A) [21, 22].

To cleave the N-glycans bound to the two plasma fibronectins, 500 μg of purified plasma fibronectin was incubated with 3 units of N-glycosidase F (PNGase F) (Roche) in a total volume of 1 ml overnight at 37°C. To catalyze the hydrolysis of α2-3, α2-6, and α2-8 linked N-acetyl-neuraminic acid residues, 100 μg purified plasma fibronectin was incubated with 100 units of α2-3,6,8 Neuraminidase (NEB) in a total volume of 1 ml overnight at 37°C.

### Glycopeptide identification for plasma fibronectin

Precipitated FN protein pellets (∼10 μg) were subjected to protein digestion using a protocol described previously [46]. The digested peptide mixtures were dissolved in 0.1 % formic acid and then analyzed using a Dionex Ultimate 3000 nanoLC system (Thermo Scientific) interfaced to an Orbitrap Fusion Tribrid mass spectrometer (Thermo Scientific) equipped with a PicoView nanosprayer (New Objective). The peptides were loaded directly onto a 25 cm x 75 μm C18 column (Acclaim PepMap® RSLC, Thermo Scientific) and separated using a 120-min linear gradient of 100% mobile phase A (0.1% formic acid in water) to 40% mobile phase B (acetonitrile with 0.1% formic acid) at a flow rate of 300nL/min. The eluted peptides were detected in the positive ion mode using a nanospray source. The mass spectrometer was programmed in the data-dependent mode over 3 sec, which consisted of a cycle of one full-scan mass spectrum (400-2000 m/z) on the Orbitrap scan with 120,000 resolution at m/z 400 and an automatic gain control (AGC) target at 200,000 followed by quadrupole isolation with higher-energy collisional dissociation (HCD) MS^2^ at a normalized collision energy of 30%. HCD MS^2^ fragment ions detected in the Orbitrap analyzer at 30,000 resolution (AGC target at 100,000) with any previously selected ions dynamically excluded for 60 s. For the database search, the MS datasets for homo and porcine plasma fibronectin were performed using the Byonic™ search energy (Protein Metrics, v.2.7.4) against FN1_human or FN1_*Sus scrofa* from the Swiss Prot (Swiss Institute of Bioinformatics) database, respectively. Protein modifications were set as carbamidomethyl (C) (fixed), deamidated (N) (variable), oxidation (M) (variable) and N-glycan modifications (182 in homo N-Glycan database; 309 in mammalian N-Glycan database). Up to two missed cleavage was allowed. The mass tolerance was set as ± 5 ppm for the MS spectra and ± 10 ppm for the MS/MS spectra. For glycopeptide identification, the Byonic score was over 100 and the false discovery rate (FDR) was less than 1%.

### Immunofluorescence staining and image analysis

For paxillin and phalloidin staining, the cells were fixed and immunostained using a method previously described [47]. For TIRFM imaging, the cells were mounted on slides with PBS containing N-propyl gallate. TIRFM images were obtained using 60X 1.40NA or 100X 1.49NA (Oil-Immersion) Plan objective lens (Nikon) using the *iLas* multi-modal of the TIRF (Roper)/spinning disk confocal (CSUX1, Yokogawa) microscope system equipped with a Evolve EMCCD camera (Photometrics). For confocal imaging, the cells were mounted on slides with fluorescent mounting medium (Dako). Confocal images were obtained with 60X 1.40NA or 100X 1.49NA (Oil-Immersion) Plan objective lens (Nikon) using the *iLas* multi-modal of TIRF (Roper)/spinning disk confocal (CSUX1, Yokogawa) microscope system equipped with a Coolsnap HQ2 CCD camera (Photometrics).

To determine the adhesion area, TIRFM images of paxillin-stained cells were thresholded to highlight only the FAs and the areas of these regions were recorded using Metamorph [47]. The total area of recorded FAs was summed to give the adhesion area. The results are presented graphically using Excel software (Microsoft). To determine the F-actin distribution, custom scripts written in MATLAB (Mathworks) were used to quantify the fluorescence intensity of F-actin along the highlighted line across the confocal image of the phalloidin-stained cells and the results are presented as a graph.

### Cell spreading assay and image analysis

Cells growing on tissue culture plates were trypsinized and re-seeded on plates coated with the indicated concentration of homo or porcine plasma fibronectin to allow them to adhere and spread (30 min for U2OS, Hela and HFF1 cells). Next the cells were fixed with 4% paraformaldehyde in PBS for 20 min at room temperature and then imaged using a microscope (Eclipse TS100; Nikon) coupled with a 20X 0.45NA objective lens (Nikon) and a WHITE CCD camera operated by ISCapture software (TUCSEN). To calculate the cell spreading area, the cell area was manually circled on the phase images using Metamorph image analysis software (Molecular Device) and the results are presented graphically using Excel software (Microsoft).

### Circular dichroism spectroscopy

CD spectra were recorded using a Jasco J-810 spectropolarimeter. Scans were taken between 190 nm and 250 nm with data collecting interval of 0.1 nm. The collected dataset was averaged using ten measurements. Samples were measured at 25 °C. A 1.0 mm cuvette was used.

### Turbidity test

The turbidities of the proteins samples were tested by measuring the light transmittance at 600 nm wavelength using a Spectrophotometer DU-800 (Beckman).

### Adhesion assay

The cell adhesion assays used 96-well plates that had been pretreated with 1% denatured BSA at 37°C for 1 h and then coated with the indicated concentration of homo or porcine plasma fibronectin. For the lectin blocking experiments, the plates coated with plasma fibronectin were further coated with 40 μg/ml Aleuria Aurantia (AAL; VECTOR) or Wheat Germ Agglutinin (WGA; VECTOR) at 4°C overnight in order to block the fucose or N-acetylglucosamine present on the fibronectin, respectively. To perform the experiments, U2OS cells growing on tissue culture plates were trypsinized, re-suspended in serum-free medium and then re-seeded on the pre-treated 96-well plates for 10 min or overnight (∼ 16h). After incubation, any non-attached cells were removed completely by washing with PBS twice, and adherent cells were fixed with 5% glutaldehyde in H_2_O for 25 min at room temperature, flowed by staining with 0.1 % crystal violet in H_2_O for 25 min at room temperature. After removing any un-bound crystal violet, the crystal violet-labelled adherent cells were solubilized in 50 μl solution A (50 % ethanol and 0.1 % acetic acid in H_2_O), and the amount of crystal violet present measured using a Thermo Scientific Multiskan Spectrum at OD 550 nm. The results are presented graphically using Excel software (Microsoft). For the experiments using EDTA or Mn^2+^, the U2OS cells were re-suspended in serum-free medium containing 0.5 mM EDTA or culture medium containing 2 mM MnCl_2_, respectively.

### Wound healing analysis

U2OS, Hela or HFF1 cells growing on tissue culture plates were trypsinized and re-seeded on 10 μg/ml fibronectin-coated 6-well plates in the culture medium for 16 h and then placed in the temperature-controlled and CO_2_-controlled chamber of a microscope (Axio Observer.Z1, Zeiss) equipped with a 10X 0.25 NA objective lens (Zeiss). Time-lapse images were obtained at 15-min intervals over 12 h using an AxioCamMR3 CCD camera operated by the Zen image analysis software (Zeiss). To calculate the percentage of wound closure, the wound area over a 6-h period or a 12-h period of migration was obtained from the time-lapse movies using the Metamorph image analysis software (Molecular Device), and calculated as the ratio of net wound-healing area to the wound area at 0-h after wounding.

### Micropatterned substrate generation

Micropatterned substrates were created by a direct microcontact printing method as previously described [23-25].

### Statistical analysis

Statistical significance was measured using Student’s t-test.

## SUPPLEMENTARY MATERIALS TABLES AND FIGURES









## Abbreviation

ECM: extracellular matrix
FA: focal adhesion
CD: the circular dichroism analysis

## References

[1] Singer AJ, Clark RA (1999). Cutaneous wound healing. N Engl J Med.

[2] To WS, Midwood KS (2011). Plasma and cellular fibronectin: distinct and independent functions during tissue repair. Fibrogenesis Tissue Repair.

[3] Lariviere B, Rouleau M, Picard S, Beaulieu AD (2003). Human plasma fibronectin potentiates the mitogenic activity of platelet-derived growth factor and complements its wound healing effects. Wound Repair Regen.

[4] Burridge K, Fath K, Kelly T, Nuckolls G, Turner C (1988). Focal adhesions: transmembrane junctions between the extracellular matrix and the cytoskeleton. Annu Rev Cell Biol.

[5] Hynes RO (2002). Integrins: bidirectional, allosteric signaling machines. Cell.

[6] Jockusch BM, Bubeck P, Giehl K, Kroemker M, Moschner J, Rothkegel M, Rudiger M, Schluter K, Stanke G, Winkler J (1995). The molecular architecture of focal adhesions. Annu Rev Cell Dev Biol.

[7] Schwartz MA, Schaller MD, Ginsberg MH (1995). Integrins: emerging paradigms of signal transduction. Annu Rev Cell Dev Biol.

[8] Zaidel-Bar R, Itzkovitz S, Ma’ayan A, Iyengar R, Geiger B (2007). Functional atlas of the integrin adhesome. Nat Cell Biol.

[9] Gupton SL, Waterman-Storer CM (2006). Spatiotemporal feedback between actomyosin and focal-adhesion systems optimizes rapid cell migration. Cell.

[10] Friedl P, Wolf K (2003). Tumour-cell invasion and migration: diversity and escape mechanisms. Nat Rev Cancer.

[11] Lauffenburger DA, Horwitz AF (1996). Cell migration: a physically integrated molecular process. Cell.

[12] Webb DJ, Parsons JT, Horwitz AF (2002). Adhesion assembly, disassembly and turnover in migrating cells -- over and over and over again. Nat Cell Biol.

[13] Johnson KJ, Sage H, Briscoe G, Erickson HP (1999). The compact conformation of fibronectin is determined by intramolecular ionic interactions. J Biol Chem.

[14] Mao Y, Schwarzbauer JE (2005). Fibronectin fibrillogenesis, a cell-mediated matrix assembly process. Matrix Biol.

[15] Uitto J, Olsen DR, Fazio MJ (1989). Extracellular matrix of the skin: 50 years of progress. J Invest Dermatol.

[16] Grinnell F, Billingham RE, Burgess L (1981). Distribution of fibronectin during wound healing *in vivo*. J Invest Dermatol.

[17] Igisu K (1986). The role of fibronectin in the process of wound healing. Thromb Res.

[18] Moretti FA, Chauhan AK, Iaconcig A, Porro F, Baralle FE, Muro AF (2007). A major fraction of fibronectin present in the extracellular matrix of tissues is plasma-derived. J Biol Chem.

[19] Clark RA, Lanigan JM, DellaPelle P, Manseau E, Dvorak HF, Colvin RB (1982). Fibronectin and fibrin provide a provisional matrix for epidermal cell migration during wound reepithelialization. J Invest Dermatol.

[20] Donaldson DJ, Mahan JT (1983). Fibrinogen and fibronectin as substrates for epidermal cell migration during wound closure. J Cell Sci.

[21] Speziale P, Visai L, Rindi S, Di Poto A (2008). Purification of human plasma fibronectin using immobilized gelatin and Arg affinity chromatography. Nat Protoc.

[22] Vuento M, Vaheri A (1979). Purification of fibronectin from human plasma by affinity chromatography under non-denaturing conditions. Biochem J.

[23] Chen CS, Mrksich M, Huang S, Whitesides GM, Ingber DE (1997). Geometric control of cell life and death. Science.

[24] Pirone DM, Liu WF, Ruiz SA, Gao L, Raghavan S, Lemmon CA, Romer LH, Chen CS (2006). An inhibitory role for FAK in regulating proliferation: a link between limited adhesion and RhoA-ROCK signaling. J Cell Biol.

[25] Wang YK, Yu X, Cohen DM, Wozniak MA, Yang MT, Gao L, Eyckmans J, Chen CS (2012). Bone morphogenetic protein-2-induced signaling and osteogenesis is regulated by cell shape, RhoA/ROCK, and cytoskeletal tension. Stem Cells Dev.

[26] Pierschbacher MD, Hayman EG, Ruoslahti E (1985). The cell attachment determinant in fibronectin. J Cell Biochem.

[27] Bern M, Kil YJ, Becker C (2012). Byonic: advanced peptide and protein identification software. Curr Protoc Bioinformatics.

[28] Chou HH, Takematsu H, Diaz S, Iber J, Nickerson E, Wright KL, Muchmore EA, Nelson DL, Warren ST, Varki A (1998). A mutation in human CMP-sialic acid hydroxylase occurred after the Homo-Pan divergence. Proc Natl Acad Sci U S A.

[29] Varki A (2001). Loss of N-glycolylneuraminic acid in humans: mechanisms, consequences, and implications for hominid evolution. Am J Phys Anthropol.

[30] Ghaderi D, Taylor RE, Padler-Karavani V, Diaz S, Varki A (2010). Implications of the presence of N-glycolylneuraminic acid in recombinant therapeutic glycoproteins. Nat Biotechnol.

[31] Varki A (2010). Colloquium paper: uniquely human evolution of sialic acid genetics and biology. Proc Natl Acad Sci U S A.

[32] Paoletti AC, Parmely TJ, Tomomori-Sato C, Sato S, Zhu D, Conaway RC, Conaway JW, Florens L, Washburn MP (2006). Quantitative proteomic analysis of distinct mammalian Mediator complexes using normalized spectral abundance factors. Proc Natl Acad Sci U S A.

[33] Layne E (1957). Spectrophotometric and turbidimetric methods for measuring proteins. Methods Enzymol.

[34] Gachet C, Hanau D, Spehner D, Brisson C, Garaud JC, Schmitt DA, Ohlmann P, Cazenave JP (1993). Alpha IIb beta 3 integrin dissociation induced by EDTA results in morphological changes of the platelet surface-connected canalicular system with differential location of the two separate subunits. J Cell Biol.

[35] Gailit J, Ruoslahti E (1988). Regulation of the fibronectin receptor affinity by divalent cations. J Biol Chem.

[36] Kaji H, Yamauchi Y, Takahashi N, Isobe T (2006). Mass spectrometric identification of N-linked glycopeptides using lectin-mediated affinity capture and glycosylation site-specific stable isotope tagging. Nat Protoc.

[37] Tajiri M, Yoshida S, Wada Y (2005). Differential analysis of site-specific glycans on plasma and cellular fibronectins: application of a hydrophilic affinity method for glycopeptide enrichment. Glycobiology.

[38] Pickford AR, Smith SP, Staunton D, Boyd J, Campbell ID (2001). The hairpin structure of the (6)F1(1)F2(2)F2 fragment from human fibronectin enhances gelatin binding. EMBO J.

[39] Liu T, Qian WJ, Gritsenko MA, Camp DG, Monroe ME, Moore RJ, Smith RD (2005). Human plasma N-glycoproteome analysis by immunoaffinity subtraction, hydrazide chemistry, and mass spectrometry. J Proteome Res.

[40] Iida R, Yasuda T, Kishi K (2007). Identification of novel fibronectin fragments detected specifically in juvenile urine. FEBS J.

[41] Jia W, Lu Z, Fu Y, Wang HP, Wang LH, Chi H, Yuan ZF, Zheng ZB, Song LN, Han HH, Liang YM, Wang JL, Cai Y (2009). A strategy for precise and large scale identification of core fucosylated glycoproteins. Mol Cell Proteomics.

[42] Wang Y, Fukuda T, Isaji T, Lu J, Gu W, Lee HH, Ohkubo Y, Kamada Y, Taniguchi N, Miyoshi E, Gu J (2015). Loss of alpha1,6-fucosyltransferase suppressed liver regeneration: implication of core fucose in the regulation of growth factor receptor-mediated cellular signaling. Sci Rep.

[43] Maheshwari G, Brown G, Lauffenburger DA, Wells A, Griffith LG (2000). Cell adhesion and motility depend on nanoscale RGD clustering. J Cell Sci.

[44] Kelm S, Schauer R, Manuguerra JC, Gross HJ, Crocker PR (1994). Modifications of cell surface sialic acids modulate cell adhesion mediated by sialoadhesin and CD22. Glycoconj J.

[45] Osumi D, Takahashi M, Miyoshi E, Yokoe S, Lee SH, Noda K, Nakamori S, Gu J, Ikeda Y, Kuroki Y, Sengoku K, Ishikawa M, Taniguchi N (2009). Core fucosylation of E-cadherin enhances cell-cell adhesion in human colon carcinoma WiDr cells. Cancer Sci.

[46] Huang IH, Hsiao CT, Wu JC, Shen RF, Liu CY, Wang YK, Chen YC, Huang CM, del Alamo JC, Chang ZF, Tang MJ, Khoo KH, Kuo JC (2014). GEF-H1 controls focal adhesion signaling that regulates mesenchymal stem cell lineage commitment. J Cell Sci.

[47] Kuo JC, Han X, Hsiao CT, Yates JR, Waterman CM (2011). Analysis of the myosin-II-responsive focal adhesion proteome reveals a role for beta-Pix in negative regulation of focal adhesion maturation. Nat Cell Biol.

[48] Varki A, Cummings RD, Aebi M, Packer NH, Seeberger PH, Esko JD, Stanley P, Hart G, Darvill A, Kinoshita T, Prestegard JJ, Schnaar RL, Freeze HH (2015). Symbol nomenclature for graphical representations of glycans. Glycobiology.

