# Supplementary material for: Fibronectin in cell adhesion and migration via N-glycosylation
Source: Oncotarget. 2017 Aug 7;8(41):70653–68. doi: 10.18632/oncotarget.19969 (PMC5642584; doi:10.18632/oncotarget.19969)
Supplement: Supplementary file 1 [file oncotarget-08-70653-s001.pdf]

Fibronectin in cell adhesion and migration via N-glycosylation

SUPPLEMENTARY MATERIALS

Supplementary Table 1: The comparison of the amino acid sequences between homo and porcine fibronectin. .

See Supplementary File 1

Supplemental Table 2: The list of identified glycopeptides from homo plasma fibronectin.

See Supplementary File 2

Supplemental Table 3: The list of identified glycopeptides from porcine plasma fibronectin.

See Supplementary File 3

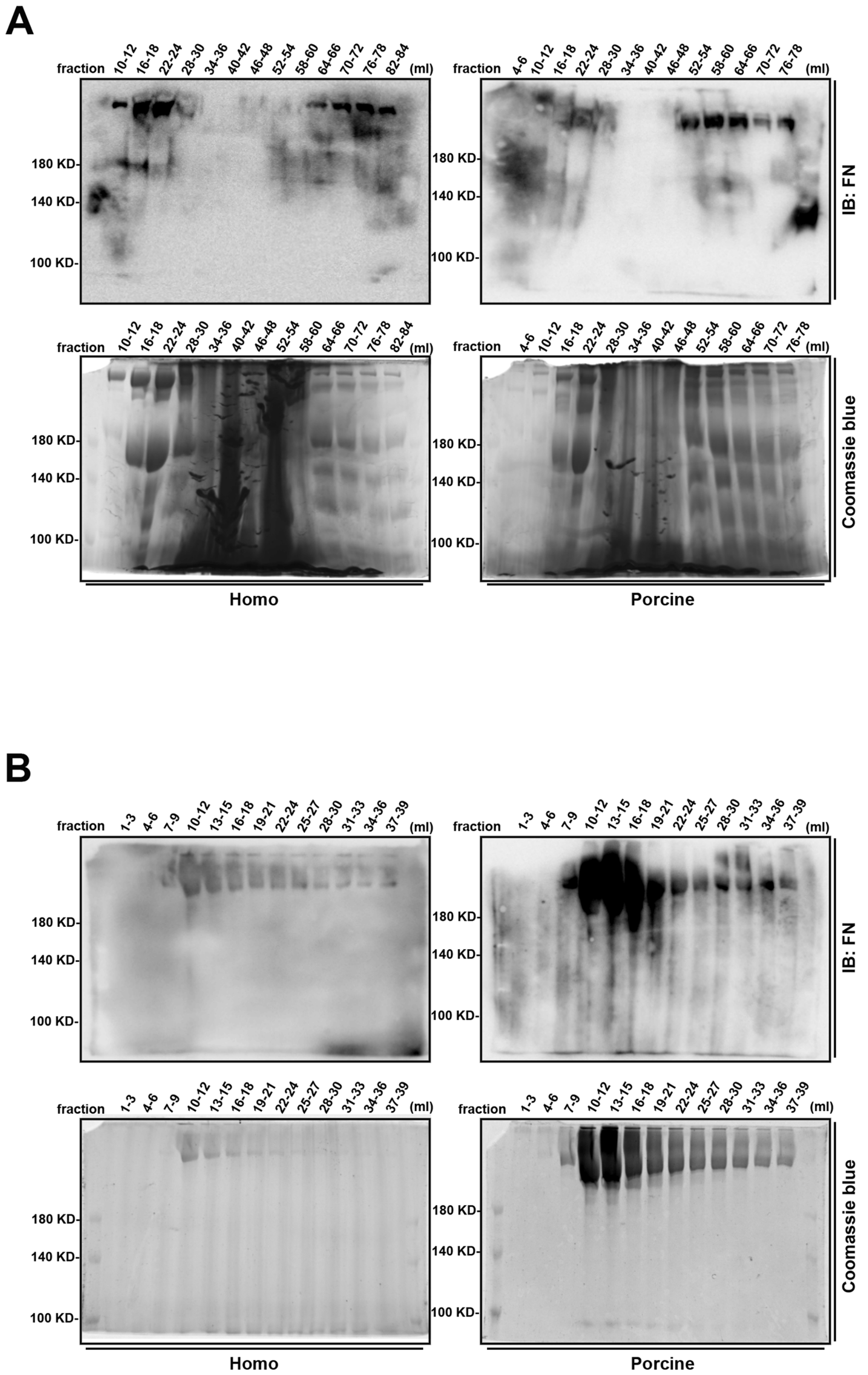

Supplementary Figure 1: Sequential separation of the two fibronectins from homo and porcine plasma. **(A)** The fractions of the flow-through materials that had passed through the Sepharose CL-4B column were analyzed by Western blotting using antibodies against fibronectin (FN) and by Coomassie blue staining. **(B)** The fractions of the eluted materials that obtained from the gelatin-Sepharose Fast Flow 4B column using 1M Arginine were analyzed by Western blotting using antibodies against fibronectin (FN) and by Coomassie blue staining.

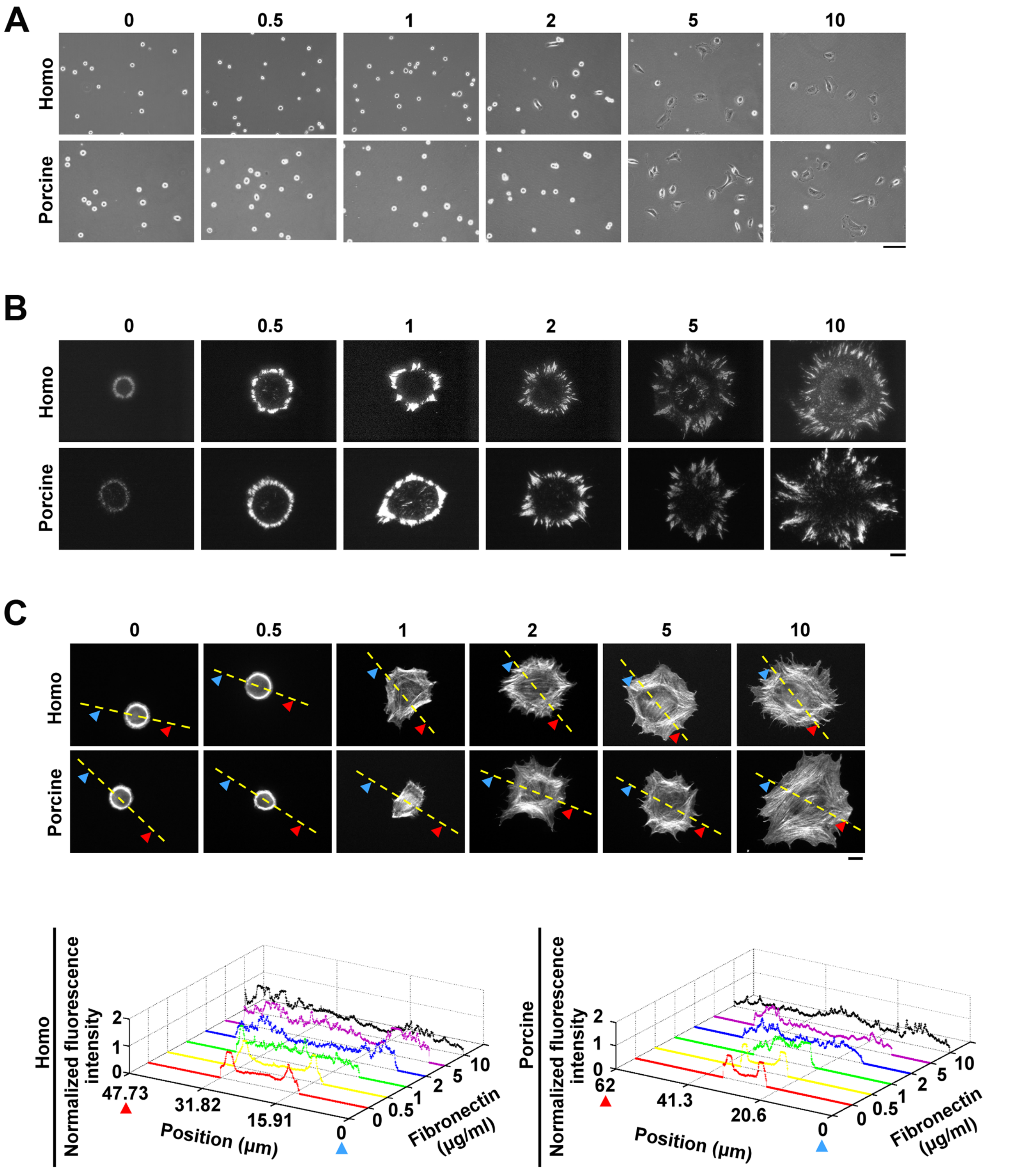

Supplementary Figure 2: Cell adhesion in response to homo and porcine plasma fibronectin using HeLa cells. **(A)** HeLa cells 30 min after plating on coverslips coated with the indicated fibronectin concentration (µg/ml). Bar, 100 µm. **(B)** TIRFM images of the HeLa cells 1.5 h after plating on coverslips coated with the indicated fibronectin concentration immunostained with paxillin. Bar, 10 µm. **(C)** Confocal images of HeLa cells 1.5 h after plating on coverslips coated with the indicated fibronectin concentration (µg/ml). Bar, 10 µm. (Bottom) Relative fluorescence intensity taken along the line highlighted in the confocal image with the edge marked with arrows and distance.

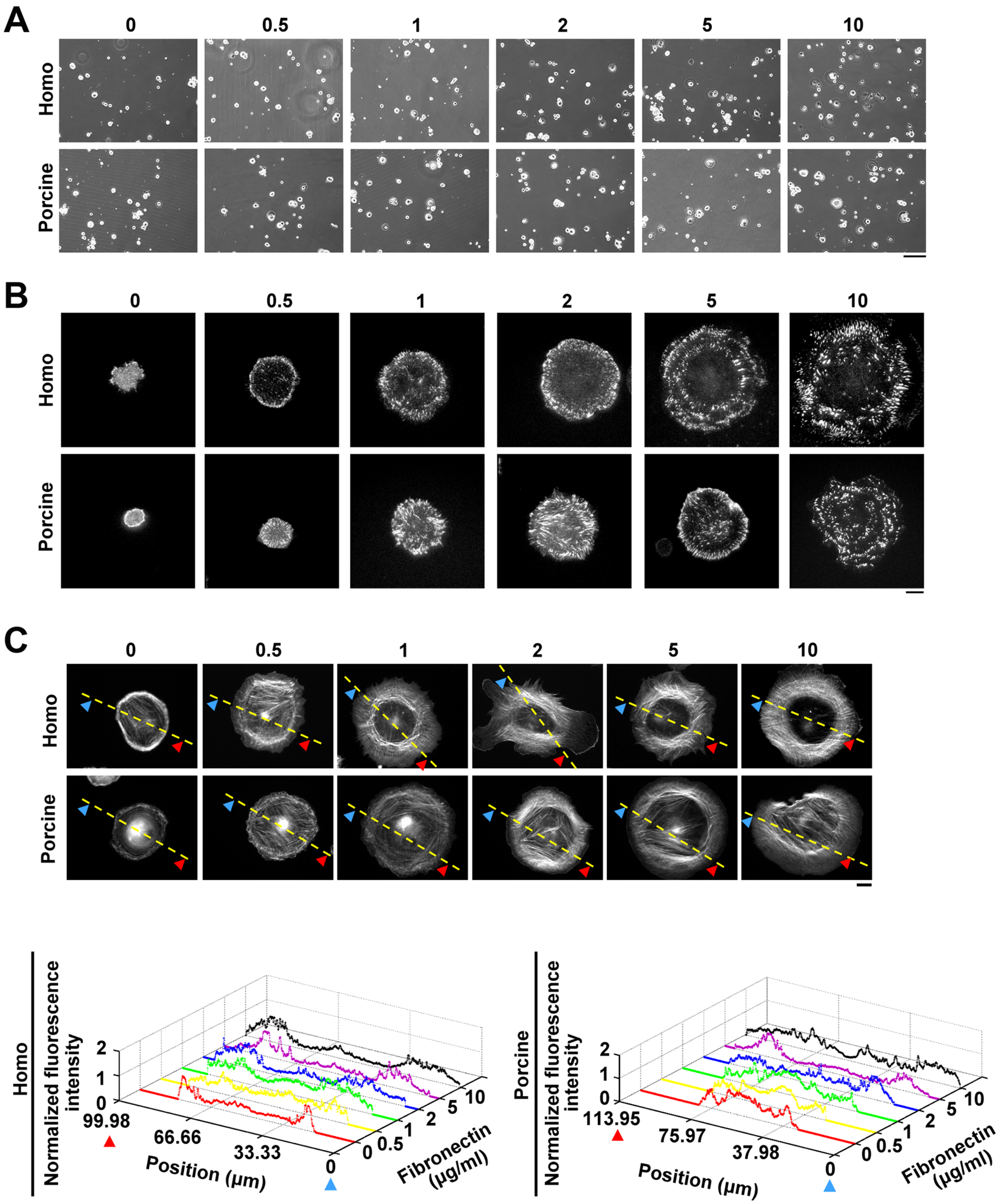

Supplementary Figure 3: Cell adhesion in response to homo and porcine plasma fibronectin using HFF1 cells. (A) HFF1 cells 30 min after plating on coverslips coated with the indicated fibronectin concentration (μg/ml). Bar, 100 μm. **(B)** TIRFM images of HFF1 cells 1.5 h after plating on coverslips coated with the indicated fibronectin concentration immunostained with paxillin. Bar, 20 μm. **(C)** Confocal images of HFF1 cells 1.5 h after plating on coverslips coated with the indicated fibronectin concentration (μg/ml). Bar, 10 (μm. (Bottom) Relative fluorescence intensity taken along the line highlighted in the confocal image with the edge marked with arrows and distance.

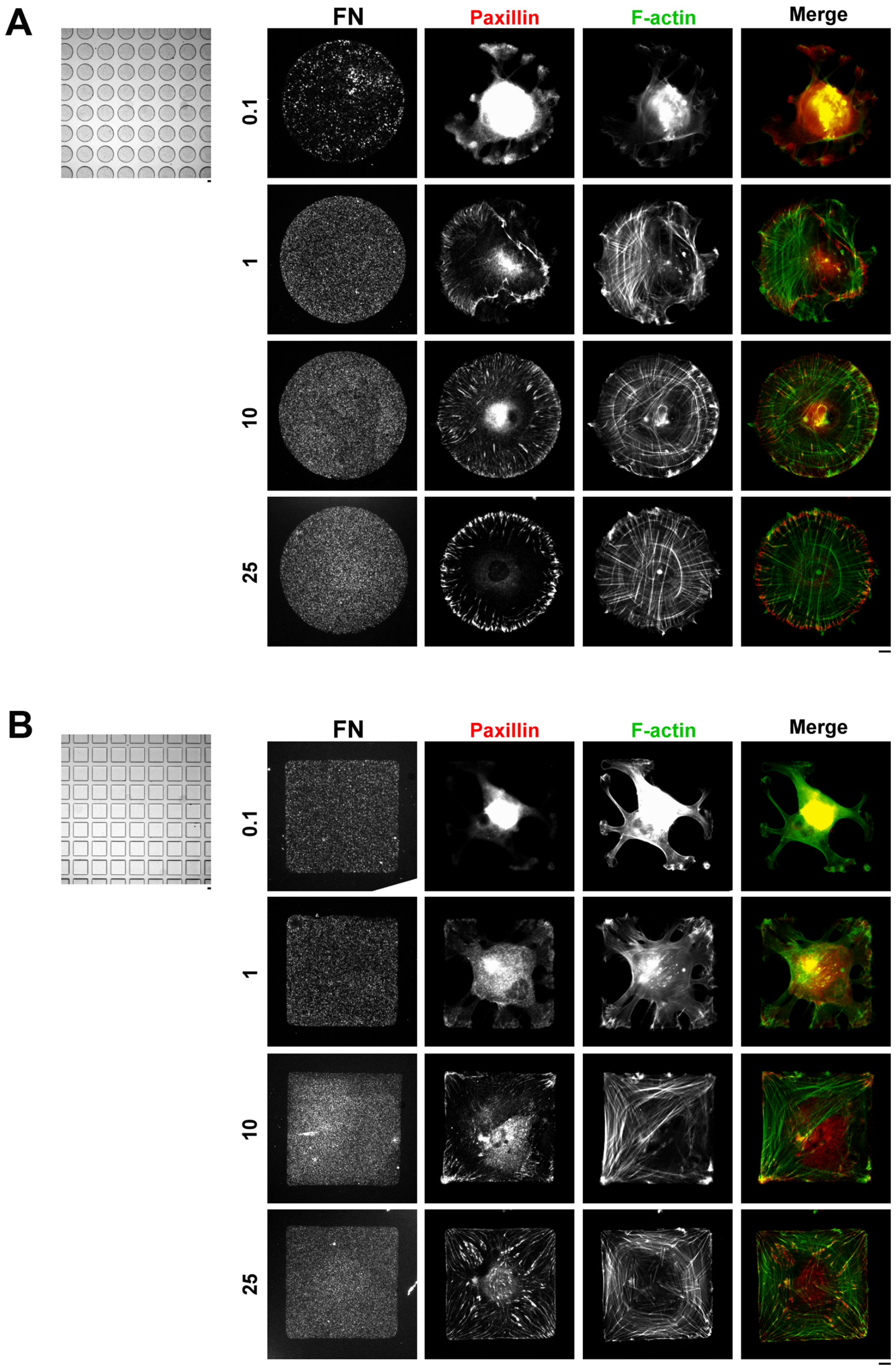

Supplementary Figure 4: Cell adhesion in response to homo and porcine plasma fibronectin using various different pattern shapes. **(A)** Confocal images of mesenchymal stem cells that were spreading on the circular micropattern (10,000 (μm^2) coated with the indicated fibronectin concentration (μg/ml), and stained with paxillin (red) and F-actin (green). Bar, 10 μm. **(B)** Confocal images of the mesenchymal stem cells that had spread on the square micropattern (10,000 μm^2) coated with the indicated fibronectin concentration (μg/ml), and stained with paxillin (red) and F-actin (green). Bar, 10 μm.

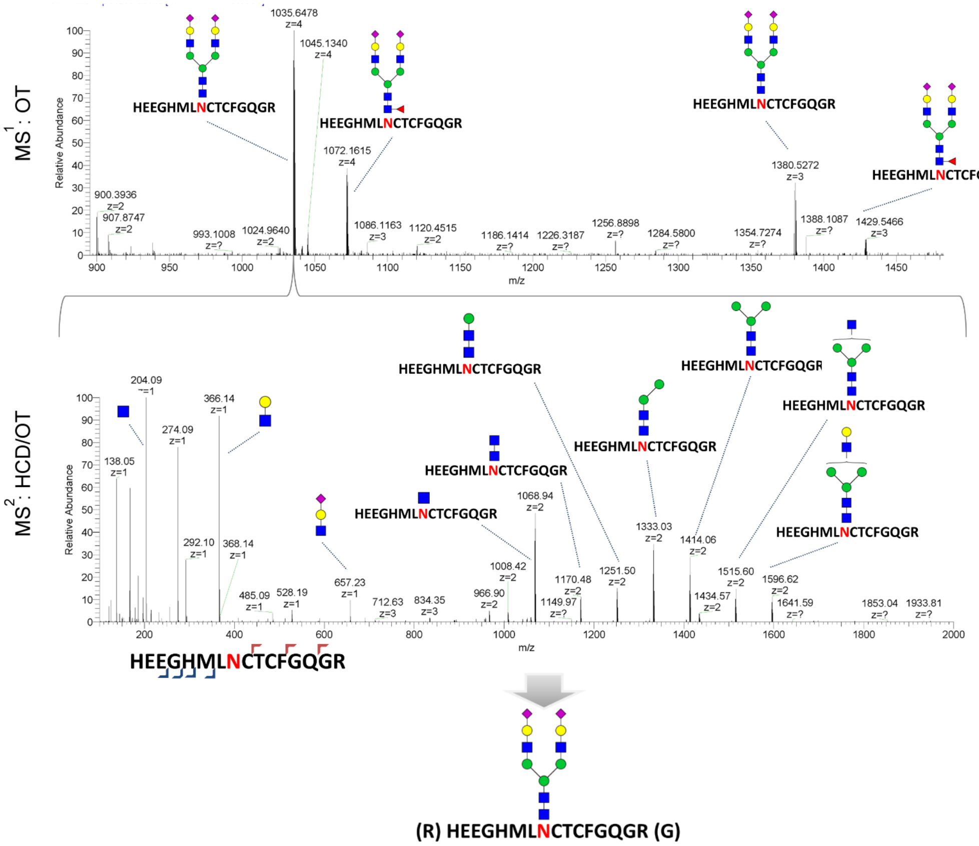

Supplementary Figure 5: LC-MS/MS-based glycopeptide sequencing and identification. (Top) A combine MS profile from scans acquired across the elution time span shows two different glycoforms, with their *m/z* values and charge states in the spectrum magnified for the mass range *m/z* 900-1500. (Bottom) A glycopeptide HCD-MS^2 spectrum for one identified glycoform is shown as an example. The Y1 ion at *m/z* 1068.94/2$^+$ for the peptide core contains the implicated N-glycosylation site N542, with glycan combination.
