# Supplementary material for: Fibronectin in cell adhesion and migration via N-glycosylation
Source: Oncotarget. 2017 Aug 7;8(41):70653–68. doi: 10.18632/oncotarget.19969 (PMC5642584; doi:10.18632/oncotarget.19969)
Supplement: Supplementary file 2 [file oncotarget-08-70653-s002.docx]

**Supplementary Table 1: The comparison of the amino acid sequences between homo and porcine fibronectin**

| Homo | MLRGPGPGLLLLAVQCLGTAVPSTGASKSKRQAQQMVQPQSPVAVSQSKPGCYDNGKHYQ |
| --- | --- |
| Porcine | MLGGPGPGLLLLAVLSLGTTVPSTGASKSKRQAQQIVQPQSPLVDSQRKPGCYDNGKHYQ |
|  |  |
| Homo | INQQWERTYLGNALVCTCYGGSRGFNCESKPEAEETCFDKYTGNTYRVGDTYERPKDSMI |
| Porcine | INQQWERTYLGSALVCTCYGGSRGFNCESKPEPEETCFDKYTGNTYRVGDTYERPKDSMI |
|  |  |
| Homo | WDCTCIGAGRGRISCTIANRCHEGGQSYKIGDTWRRPHETGGYMLECVCLGNGKGEWTCK |
| Porcine | WDCTCIGAGRGRISCTIANRCHEGGQSYKIGDTWRRPHETGGYMLECVCLGNGKGEWTCK |
|  |  |
| Homo | PIAEKCFDHAA-GTSYVVGETWEKPYQGWMMVDCTCLGEGSGRITCTSRNRCNDQDTRTS |
| Porcine | PIAEKCFDHAAGGTSYVVGETWEKPYQGWMMVDCTCLGEGSGRITCTSRNRCNDQDTRTS |
|  |  |
| Homo | YRIGDTWSKKDNRGNLLQCICTGNGRGEWKCERHTSVQTTSSGSGPFTDVRAAVYQPQPH |
| Porcine | YRIGDTWSKKDNRGNLLQCICTGNGRGEWKCERHTSLQTTSAGSGSFTDVRTAIYQPQPH |
|  |  |
| Homo | PQPPPYGHCVTDSGVVYSVGMQWLKTQGNKQMLCTCLGNGVSCQETAVTQTYGGNSNGEP |
| Porcine | PQPAPYGHCVTDSGVVYSVGMQWLKTQGNKQMLCTCLGNGVSCQETAVTQTYGGNSNGEP |
|  |  |
| Homo | CVLPFTYNGRTFYSCTTEGRQDGHLWCSTTSNYEQDQKYSFCTDHTVLVQTRGGNSNGAL |
| Porcine | CVLPFTYNGRTFYSCTTEGRQDGHLWCSTTSNYEQDQKYSFCTDHTVLVQTRGGNSNGAL |
|  |  |
| Homo | CHFPFLYNNHNYTDCTSEGRRDNMKWCGTTQNYDADQKFGFCPMAAHEEICTTNEGVMYR |
| Porcine | CHFPFLYNNRNYTDCTSEGRRDNMKWCGTTQNYDADQKFGFCPMAAHEEICTTNEGVMYR |
|  |  |
| Homo | IGDQWDKQHDMGHMMRCTCVGNGRGEWTCIAYSQLRDQCIVDDITYNVNDTFHKRHEEGH |
| Porcine | IGDQWDKQHDMGHMMRCTCVGNGRGEWTCVAYSQLRDQCIVDDITYNVNDTFHKRHEEGH |
|  |  |
| Homo | MLNCTCFGQGRGRWKCDPVDQCQDSETGTFYQIGDSWEKYVHGVRYQCYCYGRGIGEWHC |
| Porcine | MLNCTCFGQGRGRWKCDPVDQCQDSETRTFYQIGDSWEKYVHGVRYQCYCYGRGIGEWHC |
|  |  |
| Homo | QPLQTYPSSSGPVEVFITETPSQPNSHPIQWNAPQPSHISKYILRWRPKNSVGRWKEATI |
| Porcine | QPLQTYPGTTGPVQVIITETPSQPNSHPIQWNAPEPSHISKYILRWKPKNSPNRWKEATI |
|  |  |
| Homo | PGHLNSYTIKGLKPGVVYEGQLISIQQYGHQEVTRFDFTTTSTSTPVTSNTVTGETTPFS |
| Porcine | PGHLNSYTIKGLRPGVVYEGQLISVQHYGHREVTRFDFTTTSTSSAVTSNTVVGETTPFS |
|  |  |
| Homo | PLVATSESVTEITASSFVVSWVSASDTVSGFRVEYELSEEGDEPQYLDLPSTATSVNIPD |
| Porcine | PVVATSESVTEITASSFVVSWVSASDTVSGFRVEYELSEEGDEPQYLDLPSTATSVNIPD |
|  |  |
| Homo | LLPGRKYIVNVYQISEDGEQSLILSTSQTTAPDAPPDTTVDQVDDTSIVVRWSRPQAPIT |
| Porcine | LLPGRKYIVNVYQISEEGEQSLILSTSQTTAPDAPPDPTVDQVDDTSIVVRWSRPQAPIT |
|  |  |
| Homo | GYRIVYSPSVEGSSTELNLPETANSVTLSDLQPGVQYNITIYAVEENQESTPVVIQQETT |
| Porcine | GYRIVYSPSVEGSSTELNLPETANSVTLSDLQPGVQYNITIYAVEENQESTPVFIQQETT |
|  |  |
| Homo | GTPRSDTVPSPRDLQFVEVTDVKVTIMWTPPESAVTGYRVDVIPVNLPGEHGQRLPISRN |
| Porcine | GVPRPDKVPPPKDLQFVEVTDVKVTIMWTPPESPVTGYRVDVIPVNLPGEHGQRLPISRN |
|  |  |
| Homo | TFAEVTGLSPGVTYYFKVFAVSHGRESKPLTAQQTTKLDAPTNLQFVNETDSTVLVRWTP |
| Porcine | TFAEVTGLSPGVTYHFKVFAVNQGRESKPLTAQQTTKLDAPTNLQFINETDSTVMVTWTP |
|  |  |
| Homo | PRAQITGYRLTVGLTRRGQPRQYNVGPSVSKYPLRNLQPASEYTVSLVAIKGNQESPKAT |
| Porcine | PRARIAGYRLTVGLTRGGQPKQYNVGPSASQYLLRNLQPGSEYAVTLVAVKGNQQSPRAT |
|  |  |
| Homo | GVFTTLQPGSSIPPYNTEVTETTIVITWTPAPRIGFKLGVRPSQGGEAPREVTSDSGSIV |
| Porcine | GVFTTLQPVGSIPPYNTEVTETTIVITWTPAPRIGFKLGVRPSQGGEAPREVTSDSGSIV |
|  |  |
| Homo | VSGLTPGVEYVYTIQVLRDGQERDAPIVNKVVTPLSPPTNLHLEANPDTGVLTVSWERST |
| Porcine | VSGLTPGVEYVYTISVLRDGQERDTPIVKKVVTPLSPPTNLHLEANPDTGVLTVSWERST |
|  |  |
| Homo | TPDITGYRITTTPTNGQQGNSLEEVVHADQSSCTFDNLSPGLEYNVSVYTVKDDKESVPI |
| Porcine | TPDITGYRITTTPTNGQQGYSLEEVVHADQSSCTFENLSPGLEYNVSVYTVKNDKESVPI |
|  |  |
| Homo | SDTIIPEVPQLTDLSFVDITDSSIGLRWTPLNSSTIIGYRITVVAAGEGIPIFEDFVDSS |
| Porcine | SDTIIPEVPQLTDLSFVDITDSSIGLRWTPINSSTIIGYRITVVAAGEGIPIFEDFADSS |
|  |  |
| Homo | VGYYTVTGLEPGIDYDISVITLINGGESAPTTLTQQTAVPPPTDLRFTNIGPDTMRVTWA |
| Porcine | VGYYTVTGLEPGIDYDISVITLINGGESAPTTLTQQTAVPPPTDLRFTNVGPDTIRVTWA |
|  |  |
| Homo | PPPSIDLTNFLVRYSPVKNEEDVAELSISPSDNAVVLTNLLPGTEYVVSVSSVYEQHEST |
| Porcine | PPPSIELTNFLVRYSPVKNEEDVAELSISPSDNAVVLTNLLPGTEYLVSVSSVYEQHESI |
|  |  |
| Homo | PLRGRQKTGLDSPTGIDFSDITANSFTVHWIAPRATITGYRIRHHPEHFSGRPREDRVPH |
| Porcine | PLRGRQKTGLDSPTGIDFSDITANSFTVYWIAPRATITGYKIRHHPEHMGGRPREDRVPP |
|  |  |
| Homo | SRNSITLTNLTPGTEYVVSIVALNGREESPLLIGQQSTVSDVPRDLEVVAATPTSLLISW |
| Porcine | SRNSITLTNLIPGVEYVVSIVAVNGREESPPLVGQQSTVSDVPRDLQVIATTPTSLLISW |
|  |  |
| Homo | DAPAVTVRYYRITYGETGGNSPVQEFTVPGSKSTATISGLKPGVDYTITVYAVTGRGDSP |
| Porcine | DAPAVTVRYYRITYGETGGNSPVQEFTVPGSKSTATISGLKPGVDYTITVYAVTGRGDSP |
|  |  |
| Homo | ASSKPISINYRTEIDKPSQMQVTDVQDNSISVKWLPSSSPVTGYRVTTTPKNGPGPTKTK |
| Porcine | ASSKPVSIDYRTEIDKPSQMQVTDVQDNSISVRWLPSSSHVTGYRVTTTPKNGSGPSKTK |
|  |  |
| Homo | TAGPDQTEMTIEGLQPTVEYVVSVYAQNPSGESQPLVQTAVTNIDRPKGLAFTDVDVDSI |
| Porcine | TVGPDQTEMTIEGLQPTVEYVVSVYAQNQNGESQPLVQTAVTNIDRPKGLAFTDVDVDSI |
|  |  |
| Homo | KIAWESPQGQVSRYRVTYSSPEDGIHELFPAPDGEEDTAELQGLRPGSEYTVSVVALHDD |
| Porcine | KIAWESPQGQVSRYRVTYSSPEDGIHELFPAPDGEEDTAELQGLRPGSEYTVSVVALHDD |
|  |  |
| Homo | MESQPLIGTQSTAIPAPTDLKFTQVTPTSLSAQWTPPNVQLTGYRVRVTPKEKTGPMKEI |
| Porcine | MESQPLIGTQSTAIPAPTNLKFTQVTPTSLTAQWTAPNVQLTGYRVRVTPKEKTGPMKEI |
|  |  |
| Homo | NLAPDSSSVVVSGLMVATKYEVSVYALKDTLTSRPAQGVVTTLENVSPPRRARVTDATET |
| Porcine | NLAPDSSSVVVSGLMVATKYEVSIYALKDTLTSRPAQGVVTTLENVSPPRRARVTDATET |
|  |  |
| Homo | TITISWRTKTETITGFQVDAVPANGQTPIQRTIKPDVRSYTITGLQPGTDYKIYLYTLND |
| Porcine | TITISWRTKTETITGFQVDAVPANGQTPIQRTIKPDVRSYTITGLQPGTDYKIYLYTLND |
|  |  |
| Homo | NARSSPVVIDASTAIDAPSNLRFLATTPNSLLVSWQPPRARITGYIIKYEKPGSPPREVV |
| Porcine | NARSSPVVIDASTAIDAPSNLRFLATTPNSLLVSWQPPRAKITGYIIKYEKPGSPPREVV |
|  |  |
| Homo | PRPRPGVTEATITGLEPGTEYTIYVIALKNNQKSEPLIGRKKTDELPQLVTLPHPNLHGP |
| Porcine | PRPRPGVTEATITGLEPATEYTIQVIALKNNQKSEPLIGRKRTDELPQLVTLPHPNLHGP |
|  |  |
| Homo | EILDVPSTVQKTPFVTHPGYDTGNGIQLPGTSGQQPSVGQQMIFEEHGFRRTTPPTTATP |
| Porcine | EILDVPSTVQKTPFVTKPGYDTGNGIQLPGTSGQQPSLGQQMIFEEHGFRRTTPPTTATP |
|  |  |
| Homo | IRHRPRPYPPNVGEEIQIGHIPREDVDYHLYPHGPGLNPNASTGQEALSQTTISWAPFQD |
| Porcine | VRHRPGPYTPNVNEEIQVGHVPRGDVDHHLYPHVLGLNPNASTGQEALSQTTISWTPFQE |
|  |  |
| Homo | TSEYIISCHPVGTDEEPLQFRVPGTSTSATLTGLTRGATYNVIVEALKDQQRHKVREEVV |
| Porcine | SSEYIISCHPVGIDEEPLQFRVPGTSASATLTGLTRGATYNIIVEALKDQKRHKIREEVV |
|  |  |
| Homo | TVGNSVNEGLNQPTDDSCFDPYTVSHYAVGDEWERMSESGFKLLCQCLGFGSGHFRCDSS |
| Porcine | TVGNSVDQGLSQPTDDSCFDPYTVSHYAIGEEWERLSESGFKLSCQCLGFGSGHFRCDSS |
|  |  |
| Homo | RWCHDNGVNYKIGEKWDRQGENGQMMSCTCLGNGKGEFKCDPHEATCYDDGKTYHVGEQW |
| Porcine | KWCHDNGVNYKIGEKWDRQGENGQMMSCTCLGNGKGEFKCDPHEATCYDDGKTYHVGEQW |
|  |  |
| Homo | QKEYLGAICSCTCFGGQRGWRCDNCRRPGGEPSPEGTTGQSYNQYSQRYHQRTNTNVNCP |
| Porcine | QKEYLGAICSCTCFGGQRGWRCDNCRRPGAELGPEGSTGHSYNQYSQRYHQRTNTNVNCP |
|  |  |
| Homo | IECFMPLDVQADREDSRE |
| Porcine | IECFMPLDVQADIEDSRE |
|  |  |

Mismatched amino acid sequences are shown in blue. The RGD sequence is indicated by red squares.
