# Supplementary material for: Fibronectin in cell adhesion and migration via N-glycosylation
Source: Oncotarget. 2017 Aug 7;8(41):70653–68. doi: 10.18632/oncotarget.19969 (PMC5642584; doi:10.18632/oncotarget.19969)
Supplement: Supplementary file 3 [file oncotarget-08-70653-s003.docx]

|  |  |
| --- | --- |

**Supplementary Table 2:** **The list of identified glycopeptides from homo plasma fibronectin**

| **Peptide** | **Glycans** | **Starting position** | **Score** | **Scan time** |
| --- | --- | --- | --- | --- |
| R.GGNSNGALC[+57.021]HFPFLYNNHN[+1548.545]YTDC[+57.021]TSEGR.R | HexNAc(3)Hex(4)NeuAc(1) | 412 | 802.4 | 55.1838 |
| R.GGNSNGALC[+57.021]HFPFLYNNHN[+1548.545]YTDC[+57.021]TSEGR.R | HexNAc(3)Hex(4)NeuAc(1) | 412 | 335.6 | 57.6623 |
| R.GGNSNGALC[+57.021]HFPFLYNNHN[+1548.545]YTDC[+57.021]TSEGR.R | HexNAc(3)Hex(4)NeuAc(1) | 412 | 509.2 | 57.914 |
| R.GGNSNGALC[+57.021]HFPFLYNNHN[+1548.545]YTDC[+57.021]TSEGR.R | HexNAc(3)Hex(4)NeuAc(1) | 412 | 325.0 | 110.6982 |
| R.GGNSNGALC[+57.021]HFPFLYNNHN[+1710.598]YTDC[+57.021]TSEGR.R | HexNAc(3)Hex(5)NeuAc(1) | 412 | 363.2 | 57.4288 |
| R.GGNSNGALC[+57.021]HFPFLYNNHN[+1872.651]YTDC[+57.021]TSEGR.R | HexNAc(3)Hex(6)NeuAc(1) | 412 | 556.4 | 57.3439 |
| R.GGNSNGALC[+57.021]HFPFLYNNHN[+1751.624]YTDC[+57.021]TSEGR.R | HexNAc(4)Hex(4)NeuAc(1) | 412 | 230.5 | 55.1995 |
| R.GGNSNGALC[+57.021]HFPFLYNNHN[+1751.624]YTDC[+57.021]TSEGR.R | HexNAc(4)Hex(4)NeuAc(1) | 412 | 468.4 | 56.7221 |
| R.GGNSNGALC[+57.021]HFPFLYNNHN[+1751.624]YTDC[+57.021]TSEGR.R | HexNAc(4)Hex(4)NeuAc(1) | 412 | 308.0 | 57.4492 |
| R.GGNSNGALC[+57.021]HFPFLYNNHN[+1751.624]YTDC[+57.021]TSEGR.R | HexNAc(4)Hex(4)NeuAc(1) | 412 | 504.9 | 58.8957 |
| R.GGNSNGALC[+57.021]HFPFLYNNHN[+1751.624]YTDC[+57.021]TSEGR.R | HexNAc(4)Hex(4)NeuAc(1) | 412 | 206.3 | 60.5185 |
| R.GGNSNGALC[+57.021]HFPFLYNNHN[+1768.640]YTDC[+57.021]TSEGR.R | HexNAc(4)Hex(5)Fuc(1) | 412 | 291.1 | 53.8744 |
| R.GGNSNGALC[+57.021]HFPFLYNNHN[+1913.677]YTDC[+57.021]TSEGRR.D | HexNAc(4)Hex(5)NeuAc(1) | 412 | 310.3 | 49.8596 |
| R.GGNSNGALC[+57.021]HFPFLYNNHN[+1913.677]YTDC[+57.021]TSEGRR.D | HexNAc(4)Hex(5)NeuAc(1) | 412 | 206.1 | 49.8847 |
| R.GGNSNGALC[+57.021]HFPFLYNNHN[+1913.677]YTDC[+57.021]TSEGRR.D | HexNAc(4)Hex(5)NeuAc(1) | 412 | 423.6 | 50.3254 |
| R.GGNSNGALC[+57.021]HFPFLYNNHN[+1913.677]YTDC[+57.021]TSEGRR.D | HexNAc(4)Hex(5)NeuAc(1) | 412 | 384.7 | 50.3355 |
| R.GGNSNGALC[+57.021]HFPFLYNNHN[+1913.677]YTDC[+57.021]TSEGRR.D | HexNAc(4)Hex(5)NeuAc(1) | 412 | 426.5 | 50.3367 |
| R.GGNSNGALC[+57.021]HFPFLYNNHN[+1913.677]YTDC[+57.021]TSEGR.R | HexNAc(4)Hex(5)NeuAc(1) | 412 | 616.4 | 54.8326 |
| R.GGNSNGALC[+57.021]HFPFLYNNHN[+1913.677]YTDC[+57.021]TSEGR.R | HexNAc(4)Hex(5)NeuAc(1) | 412 | 446.0 | 54.8628 |
| R.GGNSNGALC[+57.021]HFPFLYNNHN[+1913.677]YTDC[+57.021]TSEGR.R | HexNAc(4)Hex(5)NeuAc(1) | 412 | 489.7 | 54.8858 |
| R.GGNSNGALC[+57.021]HFPFLYNNHN[+1913.677]YTDC[+57.021]TSEGR.R | HexNAc(4)Hex(5)NeuAc(1) | 412 | 667.1 | 55.1262 |
| R.GGNSNGALC[+57.021]HFPFLYNNHN[+1913.677]YTDC[+57.021]TSEGR.R | HexNAc(4)Hex(5)NeuAc(1) | 412 | 243.4 | 55.8337 |
| R.GGNSNGALC[+57.021]HFPFLYNNHN[+1913.677]YTDC[+57.021]TSEGR.R | HexNAc(4)Hex(5)NeuAc(1) | 412 | 253.5 | 56.124 |
| R.GGNSNGALC[+57.021]HFPFLYNNHN[+1913.677]YTDC[+57.021]TSEGR.R | HexNAc(4)Hex(5)NeuAc(1) | 412 | 257.2 | 56.2672 |
| R.GGNSNGALC[+57.021]HFPFLYNNHN[+1913.677]YTDC[+57.021]TSEGR.R | HexNAc(4)Hex(5)NeuAc(1) | 412 | 402.3 | 57.4078 |
| R.GGNSNGALC[+57.021]HFPFLYNNHN[+1913.677]YTDC[+57.021]TSEGR.R | HexNAc(4)Hex(5)NeuAc(1) | 412 | 349.0 | 58.6367 |
| R.GGNSNGALC[+57.021]HFPFLYNNHN[+1913.677]YTDC[+57.021]TSEGR.R | HexNAc(4)Hex(5)NeuAc(1) | 412 | 260.2 | 58.6977 |
| R.GGNSNGALC[+57.021]HFPFLYNNHN[+1913.677]YTDC[+57.021]TSEGR.R | HexNAc(4)Hex(5)NeuAc(1) | 412 | 196.0 | 59.8471 |
| R.GGNSNGALC[+57.021]HFPFLYNNHN[+1913.677]YTDC[+57.021]TSEGR.R | HexNAc(4)Hex(5)NeuAc(1) | 412 | 282.8 | 110.9504 |
| R.GGNSNGALC[+57.021]HFPFLYNNHN[+2204.772]YTDC[+57.021]TSEGR.R | HexNAc(4)Hex(5)NeuAc(2) | 412 | 438.3 | 60.8097 |
| R.GGNSNGALC[+57.021]HFPFLYNNHN[+2204.772]YTDC[+57.021]TSEGR.R | HexNAc(4)Hex(5)NeuAc(2) | 412 | 145.3 | 61.8167 |
| R.GGNSNGALC[+57.021]HFPFLYNNHN[+2204.772]YTDC[+57.021]TSEGR.R | HexNAc(4)Hex(5)NeuAc(2) | 412 | 118.1 | 62.5517 |
| R.GGNSNGALC[+57.021]HFPFLYNNHN[+1825.661]YTDC[+57.021]TSEGR.R | HexNAc(5)Hex(5) | 412 | 108.5 | 60.6958 |
| R.DQC[+57.021]IVDDITYNVN[+1768.640]DTFHK.R | HexNAc(4)Hex(5)Fuc(1) | 516 | 746.0 | 57.3452 |
| R.DQC[+57.021]IVDDITYNVN[+2059.735]DTFHK.R | HexNAc(4)Hex(5)Fuc(1)NeuAc(1) | 516 | 574.6 | 61.3278 |
| R.DQC[+57.021]IVDDITYNVN[+2059.735]DTFHK.R | HexNAc(4)Hex(5)Fuc(1)NeuAc(1) | 516 | 320.0 | 61.3642 |
| R.DQC[+57.021]IVDDITYNVN[+2059.735]DTFHK.R | HexNAc(4)Hex(5)Fuc(1)NeuAc(1) | 516 | 651.1 | 61.7364 |
| R.DQC[+57.021]IVDDITYNVN[+2350.830]DTFHK.R | HexNAc(4)Hex(5)Fuc(1)NeuAc(2) | 516 | 566.4 | 66.0961 |
| R.DQC[+57.021]IVDDITYNVN[+2350.830]DTFHK.R | HexNAc(4)Hex(5)Fuc(1)NeuAc(2) | 516 | 668.4 | 66.1092 |
| R.DQC[+57.021]IVDDITYNVN[+2350.830]DTFHK.R | HexNAc(4)Hex(5)Fuc(1)NeuAc(2) | 516 | 449.2 | 66.9247 |
| R.DQC[+57.021]IVDDITYNVN[+1913.677]DTFHKR.H | HexNAc(4)Hex(5)NeuAc(1) | 516 | 710.6 | 58.7075 |
| R.DQC[+57.021]IVDDITYNVN[+1913.677]DTFHKR.H | HexNAc(4)Hex(5)NeuAc(1) | 516 | 631.0 | 58.7217 |
| R.DQC[+57.021]IVDDITYNVN[+1913.677]DTFHK.R | HexNAc(4)Hex(5)NeuAc(1) | 516 | 628.0 | 61.5691 |
| R.DQC[+57.021]IVDDITYNVN[+1913.677]DTFHK.R | HexNAc(4)Hex(5)NeuAc(1) | 516 | 753.9 | 61.5807 |
| R.DQC[+57.021]IVDDITYNVN[+1913.677]DTFHK.R | HexNAc(4)Hex(5)NeuAc(1) | 516 | 583.6 | 61.9387 |
| R.DQC[+57.021]IVDDITYNVN[+1913.677]DTFHK.R | HexNAc(4)Hex(5)NeuAc(1) | 516 | 668.5 | 62.0073 |
| R.DQC[+57.021]IVDDITYNVN[+1913.677]DTFHK.R | HexNAc(4)Hex(5)NeuAc(1) | 516 | 782.5 | 62.3194 |
| R.DQC[+57.021]IVDDITYNVN[+1913.677]DTFHK.R | HexNAc(4)Hex(5)NeuAc(1) | 516 | 662.8 | 62.3772 |
| R.DQC[+57.021]IVDDITYNVN[+1913.677]DTFHK.R | HexNAc(4)Hex(5)NeuAc(1) | 516 | 508.3 | 62.4504 |
| R.DQC[+57.021]IVDDITYNVN[+1913.677]DTFHK.R | HexNAc(4)Hex(5)NeuAc(1) | 516 | 639.0 | 63.0457 |
| R.DQC[+57.021]IVDDITYNVN[+1913.677]DTFHK.R | HexNAc(4)Hex(5)NeuAc(1) | 516 | 730.0 | 63.3088 |
| R.DQC[+57.021]IVDDITYNVN[+1913.677]DTFHK.R | HexNAc(4)Hex(5)NeuAc(1) | 516 | 788.7 | 63.5969 |
| R.DQC[+57.021]IVDDITYNVN[+1913.677]DTFHK.R | HexNAc(4)Hex(5)NeuAc(1) | 516 | 705.7 | 63.6896 |
| R.DQC[+57.021]IVDDITYNVN[+1913.677]DTFHK.R | HexNAc(4)Hex(5)NeuAc(1) | 516 | 726.9 | 64.1878 |
| R.DQC[+57.021]IVDDITYNVN[+1913.677]DTFHK.R | HexNAc(4)Hex(5)NeuAc(1) | 516 | 702.0 | 67.2673 |
| R.DQC[+57.021]IVDDITYNVN[+1913.677]DTFHK.R | HexNAc(4)Hex(5)NeuAc(1) | 516 | 673.3 | 67.432 |
| R.DQC[+57.021]IVDDITYNVN[+1913.677]DTFHK.R | HexNAc(4)Hex(5)NeuAc(1) | 516 | 514.6 | 67.697 |
| R.DQC[+57.021]IVDDITYNVN[+1913.677]DTFHK.R | HexNAc(4)Hex(5)NeuAc(1) | 516 | 332.8 | 68.5843 |
| R.DQC[+57.021]IVDDITYNVN[+1913.677]DTFHK.R | HexNAc(4)Hex(5)NeuAc(1) | 516 | 472.0 | 69.0837 |
| R.DQC[+57.021]IVDDITYNVN[+2204.772]DTFHKR.H | HexNAc(4)Hex(5)NeuAc(2) | 516 | 546.8 | 62.6738 |
| R.DQC[+57.021]IVDDITYNVN[+2204.772]DTFHKR.H | HexNAc(4)Hex(5)NeuAc(2) | 516 | 612.4 | 62.7153 |
| R.DQC[+57.021]IVDDITYNVN[+2204.772]DTFHKR.H | HexNAc(4)Hex(5)NeuAc(2) | 516 | 497.5 | 62.7209 |
| R.DQC[+57.021]IVDDITYNVN[+2204.772]DTFHK.R | HexNAc(4)Hex(5)NeuAc(2) | 516 | 627.4 | 66.2655 |
| R.DQC[+57.021]IVDDITYNVN[+2204.772]DTFHK.R | HexNAc(4)Hex(5)NeuAc(2) | 516 | 786.9 | 66.3696 |
| R.DQC[+57.021]IVDDITYNVN[+2204.772]DTFHK.R | HexNAc(4)Hex(5)NeuAc(2) | 516 | 687.1 | 66.7251 |
| R.DQC[+57.021]IVDDITYNVN[+2204.772]DTFHK.R | HexNAc(4)Hex(5)NeuAc(2) | 516 | 760.8 | 66.7414 |
| R.DQC[+57.021]IVDDITYNVN[+2204.772]DTFHK.R | HexNAc(4)Hex(5)NeuAc(2) | 516 | 671.6 | 67.1109 |
| R.DQC[+57.021]IVDDITYNVN[+2204.772]DTFHK.R | HexNAc(4)Hex(5)NeuAc(2) | 516 | 754.5 | 67.1462 |
| R.DQC[+57.021]IVDDITYNVN[+2204.772]DTFHK.R | HexNAc(4)Hex(5)NeuAc(2) | 516 | 633.5 | 67.5235 |
| R.DQC[+57.021]IVDDITYNVN[+2204.772]DTFHK.R | HexNAc(4)Hex(5)NeuAc(2) | 516 | 712.7 | 67.5609 |
| R.DQC[+57.021]IVDDITYNVN[+2204.772]DTFHK.R | HexNAc(4)Hex(5)NeuAc(2) | 516 | 580.3 | 67.909 |
| R.DQC[+57.021]IVDDITYNVN[+2204.772]DTFHK.R | HexNAc(4)Hex(5)NeuAc(2) | 516 | 749.7 | 67.9695 |
| R.DQC[+57.021]IVDDITYNVN[+2204.772]DTFHK.R | HexNAc(4)Hex(5)NeuAc(2) | 516 | 734.5 | 68.4003 |
| R.DQC[+57.021]IVDDITYNVN[+2204.772]DTFHK.R | HexNAc(4)Hex(5)NeuAc(2) | 516 | 308.0 | 68.4399 |
| R.DQC[+57.021]IVDDITYNVN[+2204.772]DTFHK.R | HexNAc(4)Hex(5)NeuAc(2) | 516 | 701.0 | 68.7902 |
| R.DQC[+57.021]IVDDITYNVN[+2204.772]DTFHK.R | HexNAc(4)Hex(5)NeuAc(2) | 516 | 525.7 | 68.8234 |
| R.DQC[+57.021]IVDDITYNVN[+2204.772]DTFHK.R | HexNAc(4)Hex(5)NeuAc(2) | 516 | 448.4 | 69.4297 |
| R.DQC[+57.021]IVDDITYNVN[+2204.772]DTFHK.R | HexNAc(4)Hex(5)NeuAc(2) | 516 | 682.8 | 69.6237 |
| R.DQC[+57.021]IVDDITYNVN[+2204.772]DTFHK.R | HexNAc(4)Hex(5)NeuAc(2) | 516 | 448.6 | 70.0024 |
| R.DQC[+57.021]IVDDITYNVN[+2204.772]DTFHK.R | HexNAc(4)Hex(5)NeuAc(2) | 516 | 435.4 | 70.4241 |
| R.DQC[+57.021]IVDDITYNVN[+2204.772]DTFHK.R | HexNAc(4)Hex(5)NeuAc(2) | 516 | 664.4 | 70.5681 |
| R.DQC[+57.021]IVDDITYNVN[+2204.772]DTFHK.R | HexNAc(4)Hex(5)NeuAc(2) | 516 | 611.4 | 71.012 |
| R.DQC[+57.021]IVDDITYNVN[+2204.772]DTFHK.R | HexNAc(4)Hex(5)NeuAc(2) | 516 | 283.4 | 71.1614 |
| R.DQC[+57.021]IVDDITYNVN[+2204.772]DTFHK.R | HexNAc(4)Hex(5)NeuAc(2) | 516 | 312.4 | 71.182 |
| R.DQC[+57.021]IVDDITYNVN[+2204.772]DTFHK.R | HexNAc(4)Hex(5)NeuAc(2) | 516 | 661.1 | 71.2837 |
| R.DQC[+57.021]IVDDITYNVN[+2204.772]DTFHK.R | HexNAc(4)Hex(5)NeuAc(2) | 516 | 252.3 | 71.555 |
| R.DQC[+57.021]IVDDITYNVN[+2204.772]DTFHK.R | HexNAc(4)Hex(5)NeuAc(2) | 516 | 735.7 | 71.7381 |
| R.DQC[+57.021]IVDDITYNVN[+2204.772]DTFHK.R | HexNAc(4)Hex(5)NeuAc(2) | 516 | 697.7 | 72.1891 |
| R.DQC[+57.021]IVDDITYNVN[+2204.772]DTFHK.R | HexNAc(4)Hex(5)NeuAc(2) | 516 | 227.0 | 72.2566 |
| R.DQC[+57.021]IVDDITYNVN[+2204.772]DTFHK.R | HexNAc(4)Hex(5)NeuAc(2) | 516 | 382.1 | 73.5908 |
| R.DQC[+57.021]IVDDITYNVN[+2204.772]DTFHK.R | HexNAc(4)Hex(5)NeuAc(2) | 516 | 216.3 | 76.3039 |
| R.DQC[+57.021]IVDDITYNVN[+2204.772]DTFHK.R | HexNAc(4)Hex(5)NeuAc(2) | 516 | 291.7 | 77.3546 |
| R.DQC[+57.021]IVDDITYNVN[+2204.772]DTFHK.R | HexNAc(4)Hex(5)NeuAc(2) | 516 | 539.3 | 110.5575 |
| R.DQC[+57.021]IVDDITYNVN[+2204.772]DTFHK.R | HexNAc(4)Hex(5)NeuAc(2) | 516 | 406.6 | 110.559 |
| R.DQC[+57.021]IVDDITYNVN[+1930.692]DTFHK.R | HexNAc(4)Hex(6)Fuc(1) | 516 | 149.3 | 61.6302 |
| R.DQC[+57.021]IVDDITYNVN[+2075.730]DTFHK.R | HexNAc(4)Hex(6)NeuAc(1) | 516 | 785.3 | 61.5413 |
| R.DQC[+57.021]IVDDITYNVN[+2424.867]DTFHK.R | HexNAc(5)Hex(6)Fuc(1)NeuAc(1) | 516 | 354.7 | 61.1614 |
| R.DQC[+57.021]IVDDITYNVN[+2278.809]DTFHK.R | HexNAc(5)Hex(6)NeuAc(1) | 516 | 767.1 | 61.4157 |
| R.DQC[+57.021]IVDDITYNVN[+2278.809]DTFHK.R | HexNAc(5)Hex(6)NeuAc(1) | 516 | 650.2 | 61.4183 |
| R.DQC[+57.021]IVDDITYNVN[+2278.809]DTFHK.R | HexNAc(5)Hex(6)NeuAc(1) | 516 | 765.8 | 61.4366 |
| R.DQC[+57.021]IVDDITYNVN[+2861.000]DTFHK.R | HexNAc(5)Hex(6)NeuAc(3) | 516 | 271.1 | 77.3051 |
| R.DQC[+57.021]IVDDITYNVN[+2352.846]DTFHK.R | HexNAc(6)Hex(7) | 516 | 433.9 | 66.2543 |
| K.RHEEGHMLN[+1622.582]C[+57.021]TC[+57.021]FGQGR.G | HexNAc(4)Hex(5) | 534 | 137.1 | 24.8477 |
| K.RHEEGHMLN[+1622.582]C[+57.021]TC[+57.021]FGQGR.G | HexNAc(4)Hex(5) | 534 | 229.5 | 24.8659 |
| K.RHEEGHMLN[+1768.640]C[+57.021]TC[+57.021]FGQGR.G | HexNAc(4)Hex(5)Fuc(1) | 534 | 600.4 | 24.7965 |
| K.RHEEGHMLN[+1768.640]C[+57.021]TC[+57.021]FGQGR.G | HexNAc(4)Hex(5)Fuc(1) | 534 | 264.1 | 24.8282 |
| K.RHEEGHMLN[+2059.735]C[+57.021]TC[+57.021]FGQGR.G | HexNAc(4)Hex(5)Fuc(1)NeuAc(1) | 534 | 148.6 | 26.9431 |
| K.RHEEGHMLN[+2350.830]C[+57.021]TC[+57.021]FGQGR.G | HexNAc(4)Hex(5)Fuc(1)NeuAc(2) | 534 | 237.5 | 30.6907 |
| K.RHEEGHMLN[+1913.677]C[+57.021]TC[+57.021]FGQGR.G | HexNAc(4)Hex(5)NeuAc(1) | 534 | 194.7 | 26.2668 |
| K.RHEEGHMLN[+1913.677]C[+57.021]TC[+57.021]FGQGR.G | HexNAc(4)Hex(5)NeuAc(1) | 534 | 149.3 | 27.1451 |
| K.RHEEGHMLN[+1913.677]C[+57.021]TC[+57.021]FGQGR.G | HexNAc(4)Hex(5)NeuAc(1) | 534 | 250.2 | 27.5203 |
| K.RHEEGHMLN[+1913.677]C[+57.021]TC[+57.021]FGQGR.G | HexNAc(4)Hex(5)NeuAc(1) | 534 | 492.4 | 27.5747 |
| K.RHEEGHMLN[+1913.677]C[+57.021]TC[+57.021]FGQGR.G | HexNAc(4)Hex(5)NeuAc(1) | 534 | 555.2 | 27.8714 |
| K.RHEEGHMLN[+1913.677]C[+57.021]TC[+57.021]FGQGR.G | HexNAc(4)Hex(5)NeuAc(1) | 534 | 589.4 | 27.9765 |
| K.RHEEGHMLN[+1913.677]C[+57.021]TC[+57.021]FGQGR.G | HexNAc(4)Hex(5)NeuAc(1) | 534 | 499.3 | 28.2202 |
| K.RHEEGHMLN[+1913.677]C[+57.021]TC[+57.021]FGQGR.G | HexNAc(4)Hex(5)NeuAc(1) | 534 | 607.0 | 28.2441 |
| K.RHEEGHMLN[+1913.677]C[+57.021]TC[+57.021]FGQGR.G | HexNAc(4)Hex(5)NeuAc(1) | 534 | 584.7 | 28.5801 |
| K.RHEEGHMLN[+1913.677]C[+57.021]TC[+57.021]FGQGR.G | HexNAc(4)Hex(5)NeuAc(1) | 534 | 431.3 | 28.5988 |
| K.RHEEGHMLN[+1913.677]C[+57.021]TC[+57.021]FGQGR.G | HexNAc(4)Hex(5)NeuAc(1) | 534 | 341.8 | 29.6404 |
| K.RHEEGHMLN[+2204.772]C[+57.021]TC[+57.021]FGQGR.G | HexNAc(4)Hex(5)NeuAc(2) | 534 | 345.0 | 29.4966 |
| K.RHEEGHMLN[+2204.772]C[+57.021]TC[+57.021]FGQGR.G | HexNAc(4)Hex(5)NeuAc(2) | 534 | 329.8 | 30.2691 |
| K.RHEEGHMLN[+2204.772]C[+57.021]TC[+57.021]FGQGR.G | HexNAc(4)Hex(5)NeuAc(2) | 534 | 656.8 | 30.7495 |
| K.RHEEGHMLN[+2204.772]C[+57.021]TC[+57.021]FGQGR.G | HexNAc(4)Hex(5)NeuAc(2) | 534 | 231.7 | 30.7611 |
| K.RHEEGHMLN[+2204.772]C[+57.021]TC[+57.021]FGQGR.G | HexNAc(4)Hex(5)NeuAc(2) | 534 | 171.8 | 110.8458 |
| K.RHEEGHMLN[+1930.692]C[+57.021]TC[+57.021]FGQGR.G | HexNAc(4)Hex(6)Fuc(1) | 534 | 177.0 | 23.0747 |
| K.RHEEGHMLN[+2221.788]C[+57.021]TC[+57.021]FGQGR.G | HexNAc(4)Hex(6)Fuc(1)NeuAc(1) | 534 | 256.7 | 25.5213 |
| K.RHEEGHMLN[+2352.846]C[+57.021]TC[+57.021]FGQGR.G | HexNAc(6)Hex(7) | 534 | 165.4 | 30.6623 |
| K.RHEEGHMLN[+2352.846]C[+57.021]TC[+57.021]FGQGR.G | HexNAc(6)Hex(7) | 534 | 550.9 | 30.6682 |
| R.HEEGHMLN[+1548.545]C[+57.021]TC[+57.021]FGQGR.G | HexNAc(3)Hex(4)NeuAc(1) | 535 | 938.5 | 34.9253 |
| R.HEEGHMLN[+1622.582]C[+57.021]TC[+57.021]FGQGR.G | HexNAc(4)Hex(5) | 535 | 867.0 | 29.0311 |
| R.HEEGHMLN[+1622.582]C[+57.021]TC[+57.021]FGQGR.G | HexNAc(4)Hex(5) | 535 | 429.7 | 29.0634 |
| R.HEEGHMLN[+1622.582]C[+57.021]TC[+57.021]FGQGR.G | HexNAc(4)Hex(5) | 535 | 857.4 | 31.0457 |
| R.HEEGHMLN[+1768.640]C[+57.021]TC[+57.021]FGQGR.G | HexNAc(4)Hex(5)Fuc(1) | 535 | 487.0 | 28.611 |
| R.HEEGHMLN[+1768.640]C[+57.021]TC[+57.021]FGQGR.G | HexNAc(4)Hex(5)Fuc(1) | 535 | 398.1 | 28.636 |
| R.HEEGHMLN[+1768.640]C[+57.021]TC[+57.021]FGQGR.G | HexNAc(4)Hex(5)Fuc(1) | 535 | 418.3 | 28.7009 |
| R.HEEGHMLN[+1768.640]C[+57.021]TC[+57.021]FGQGR.G | HexNAc(4)Hex(5)Fuc(1) | 535 | 510.3 | 28.7212 |
| R.HEEGHMLN[+1768.640]C[+57.021]TC[+57.021]FGQGR.G | HexNAc(4)Hex(5)Fuc(1) | 535 | 157.3 | 29.2735 |
| R.HEEGHMLN[+2059.735]C[+57.021]TC[+57.021]FGQGR.G | HexNAc(4)Hex(5)Fuc(1)NeuAc(1) | 535 | 488.9 | 30.7157 |
| R.HEEGHMLN[+2059.735]C[+57.021]TC[+57.021]FGQGR.G | HexNAc(4)Hex(5)Fuc(1)NeuAc(1) | 535 | 871.4 | 30.8122 |
| R.HEEGHMLN[+2350.830]C[+57.021]TC[+57.021]FGQGR.G | HexNAc(4)Hex(5)Fuc(1)NeuAc(2) | 535 | 436.0 | 34.2236 |
| R.HEEGHMLN[+2350.830]C[+57.021]TC[+57.021]FGQGR.G | HexNAc(4)Hex(5)Fuc(1)NeuAc(2) | 535 | 147.7 | 34.2602 |
| R.HEEGHMLN[+2350.830]C[+57.021]TC[+57.021]FGQGR.G | HexNAc(4)Hex(5)Fuc(1)NeuAc(2) | 535 | 662.4 | 34.8761 |
| R.HEEGHMLN[+1913.677]C[+57.021]TC[+57.021]FGQGR.G | HexNAc(4)Hex(5)NeuAc(1) | 535 | 686.0 | 29.6512 |
| R.HEEGHMLN[+1913.677]C[+57.021]TC[+57.021]FGQGR.G | HexNAc(4)Hex(5)NeuAc(1) | 535 | 792.9 | 29.6628 |
| R.HEEGHMLN[+1913.677]C[+57.021]TC[+57.021]FGQGR.G | HexNAc(4)Hex(5)NeuAc(1) | 535 | 561.8 | 30.8989 |
| R.HEEGHMLN[+1913.677]C[+57.021]TC[+57.021]FGQGR.G | HexNAc(4)Hex(5)NeuAc(1) | 535 | 331.7 | 31.2555 |
| R.HEEGHMLN[+1913.677]C[+57.021]TC[+57.021]FGQGR.G | HexNAc(4)Hex(5)NeuAc(1) | 535 | 910.5 | 34.7212 |
| R.HEEGHMLN[+1913.677]C[+57.021]TC[+57.021]FGQGR.G | HexNAc(4)Hex(5)NeuAc(1) | 535 | 773.4 | 34.8198 |
| R.HEEGHMLN[+1913.677]C[+57.021]TC[+57.021]FGQGR.G | HexNAc(4)Hex(5)NeuAc(1) | 535 | 790.8 | 35.0218 |
| R.HEEGHMLN[+1913.677]C[+57.021]TC[+57.021]FGQGR.G | HexNAc(4)Hex(5)NeuAc(1) | 535 | 808.9 | 35.18 |
| R.HEEGHMLN[+1913.677]C[+57.021]TC[+57.021]FGQGR.G | HexNAc(4)Hex(5)NeuAc(1) | 535 | 599.0 | 110.759 |
| R.HEEGHMLN[+2204.772]C[+57.021]TC[+57.021]FGQGR.G | HexNAc(4)Hex(5)NeuAc(2) | 535 | 221.1 | 34.513 |
| R.HEEGHMLN[+2204.772]C[+57.021]TC[+57.021]FGQGR.G | HexNAc(4)Hex(5)NeuAc(2) | 535 | 876.3 | 34.582 |
| R.HEEGHMLN[+2204.772]C[+57.021]TC[+57.021]FGQGR.G | HexNAc(4)Hex(5)NeuAc(2) | 535 | 691.4 | 34.885 |
| R.HEEGHMLN[+2204.772]C[+57.021]TC[+57.021]FGQGR.G | HexNAc(4)Hex(5)NeuAc(2) | 535 | 578.9 | 34.9632 |
| R.HEEGHMLN[+2204.772]C[+57.021]TC[+57.021]FGQGR.G | HexNAc(4)Hex(5)NeuAc(2) | 535 | 594.0 | 35.2446 |
| R.HEEGHMLN[+2204.772]C[+57.021]TC[+57.021]FGQGR.G | HexNAc(4)Hex(5)NeuAc(2) | 535 | 827.5 | 35.3321 |
| R.HEEGHMLN[+2204.772]C[+57.021]TC[+57.021]FGQGR.G | HexNAc(4)Hex(5)NeuAc(2) | 535 | 800.7 | 35.933 |
| R.HEEGHMLN[+2204.772]C[+57.021]TC[+57.021]FGQGR.G | HexNAc(4)Hex(5)NeuAc(2) | 535 | 456.6 | 36.876 |
| R.HEEGHMLN[+2204.772]C[+57.021]TC[+57.021]FGQGR.G | HexNAc(4)Hex(5)NeuAc(2) | 535 | 563.8 | 36.9887 |
| R.HEEGHMLN[+2204.772]C[+57.021]TC[+57.021]FGQGR.G | HexNAc(4)Hex(5)NeuAc(2) | 535 | 480.0 | 37.3089 |
| R.HEEGHMLN[+2204.772]C[+57.021]TC[+57.021]FGQGR.G | HexNAc(4)Hex(5)NeuAc(2) | 535 | 663.3 | 110.6842 |
| R.HEEGHMLN[+1930.692]C[+57.021]TC[+57.021]FGQGR.G | HexNAc(4)Hex(6)Fuc(1) | 535 | 331.5 | 25.5306 |
| R.HEEGHMLN[+1930.692]C[+57.021]TC[+57.021]FGQGR.G | HexNAc(4)Hex(6)Fuc(1) | 535 | 201.2 | 25.593 |
| R.HEEGHMLN[+1930.692]C[+57.021]TC[+57.021]FGQGR.G | HexNAc(4)Hex(6)Fuc(1) | 535 | 119.5 | 28.5847 |
| R.HEEGHMLN[+2221.788]C[+57.021]TC[+57.021]FGQGR.G | HexNAc(4)Hex(6)Fuc(1)NeuAc(1) | 535 | 411.1 | 29.3223 |
| R.HEEGHMLN[+2221.788]C[+57.021]TC[+57.021]FGQGR.G | HexNAc(4)Hex(6)Fuc(1)NeuAc(1) | 535 | 171.5 | 29.3573 |
| R.HEEGHMLN[+2075.730]C[+57.021]TC[+57.021]FGQGR.G | HexNAc(4)Hex(6)NeuAc(1) | 535 | 420.9 | 30.8199 |
| R.HEEGHMLN[+2092.745]C[+57.021]TC[+57.021]FGQGR.G | HexNAc(4)Hex(7)Fuc(1) | 535 | 261.7 | 41.3949 |
| R.HEEGHMLN[+1825.661]C[+57.021]TC[+57.021]FGQGR.G | HexNAc(5)Hex(5) | 535 | 358.3 | 34.1466 |
| R.HEEGHMLN[+2116.756]C[+57.021]TC[+57.021]FGQGR.G | HexNAc(5)Hex(5)NeuAc(1) | 535 | 189.8 | 37.2448 |
| R.HEEGHMLN[+2116.756]C[+57.021]TC[+57.021]FGQGR.G | HexNAc(5)Hex(5)NeuAc(1) | 535 | 309.6 | 38.054 |
| R.HEEGHMLN[+2861.000]C[+57.021]TC[+57.021]FGQGR.G | HexNAc(5)Hex(6)NeuAc(3) | 535 | 172.4 | 39.2015 |
| R.HEEGHMLN[+2149.767]C[+57.021]TC[+57.021]FGQGR.G | HexNAc(5)Hex(7) | 535 | 356.0 | 39.6039 |
| K.LDAPTNLQFVN[+1768.640]ETDSTVLVR.W | HexNAc(4)Hex(5)Fuc(1) | 997 | 618.0 | 60.9514 |
| K.LDAPTNLQFVN[+2350.830]ETDSTVLVR.W | HexNAc(4)Hex(5)Fuc(1)NeuAc(2) | 997 | 754.5 | 69.1134 |
| K.LDAPTNLQFVN[+2350.830]ETDSTVLVR.W | HexNAc(4)Hex(5)Fuc(1)NeuAc(2) | 997 | 721.8 | 69.4758 |
| K.LDAPTNLQFVN[+2350.830]ETDSTVLVR.W | HexNAc(4)Hex(5)Fuc(1)NeuAc(2) | 997 | 699.0 | 69.5921 |
| K.LDAPTNLQFVN[+1913.677]ETDSTVLVR.W | HexNAc(4)Hex(5)NeuAc(1) | 997 | 699.8 | 64.8022 |
| K.LDAPTNLQFVN[+2204.772]ETDSTVLVR.W | HexNAc(4)Hex(5)NeuAc(2) | 997 | 485.5 | 69.2355 |
| K.LDAPTNLQFVN[+2204.772]ETDSTVLVR.W | HexNAc(4)Hex(5)NeuAc(2) | 997 | 686.5 | 69.7504 |
| K.LDAPTNLQFVN[+2204.772]ETDSTVLVR.W | HexNAc(4)Hex(5)NeuAc(2) | 997 | 756.7 | 69.8294 |
| K.LDAPTNLQFVN[+2204.772]ETDSTVLVR.W | HexNAc(4)Hex(5)NeuAc(2) | 997 | 756.3 | 70.1204 |
| K.LDAPTNLQFVN[+2204.772]ETDSTVLVR.W | HexNAc(4)Hex(5)NeuAc(2) | 997 | 700.8 | 70.2074 |
| K.LDAPTNLQFVN[+2204.772]ETDSTVLVR.W | HexNAc(4)Hex(5)NeuAc(2) | 997 | 747.6 | 70.5264 |
| K.LDAPTNLQFVN[+2204.772]ETDSTVLVR.W | HexNAc(4)Hex(5)NeuAc(2) | 997 | 711.4 | 70.5972 |
| K.LDAPTNLQFVN[+2204.772]ETDSTVLVR.W | HexNAc(4)Hex(5)NeuAc(2) | 997 | 746.0 | 70.9194 |
| K.LDAPTNLQFVN[+2204.772]ETDSTVLVR.W | HexNAc(4)Hex(5)NeuAc(2) | 997 | 692.7 | 71.7023 |
| K.LDAPTNLQFVN[+2204.772]ETDSTVLVR.W | HexNAc(4)Hex(5)NeuAc(2) | 997 | 629.7 | 71.7627 |
| K.LDAPTNLQFVN[+2204.772]ETDSTVLVR.W | HexNAc(4)Hex(5)NeuAc(2) | 997 | 576.4 | 110.5559 |
| K.LDAPTNLQFVN[+2221.788]ETDSTVLVR.W | HexNAc(4)Hex(6)Fuc(1)NeuAc(1) | 997 | 362.8 | 69.7543 |
| K.LDAPTNLQFVN[+2221.788]ETDSTVLVR.W | HexNAc(4)Hex(6)Fuc(1)NeuAc(1) | 997 | 469.6 | 70.2087 |
| K.LDAPTNLQFVN[+2221.788]ETDSTVLVR.W | HexNAc(4)Hex(6)Fuc(1)NeuAc(1) | 997 | 401.1 | 70.5985 |
| K.LDAPTNLQFVN[+2075.730]ETDSTVLVR.W | HexNAc(4)Hex(6)NeuAc(1) | 997 | 430.5 | 64.7251 |
| K.LDAPTNLQFVN[+2861.000]ETDSTVLVR.W | HexNAc(5)Hex(6)NeuAc(3) | 997 | 406.6 | 79.4534 |
| K.LDAPTNLQFVN[+2861.000]ETDSTVLVR.W | HexNAc(5)Hex(6)NeuAc(3) | 997 | 557.8 | 79.8319 |
| K.LDAPTNLQFVN[+2352.846]ETDSTVLVR.W | HexNAc(6)Hex(7) | 997 | 675.3 | 69.1036 |
| K.LDAPTNLQFVN[+2352.846]ETDSTVLVR.W | HexNAc(6)Hex(7) | 997 | 699.5 | 69.5546 |
| R.ITTTPTNGQQGNSLEEVVHADQSSC[+57.021]TFDNLSPGLEYN[+1913.677]VSVYTVK.D | HexNAc(4)Hex(5)NeuAc(1) | 1208 | 531.6 | 71.997 |
| R.ITTTPTNGQQGNSLEEVVHADQSSC[+57.021]TFDNLSPGLEYN[+1913.677]VSVYTVK.D | HexNAc(4)Hex(5)NeuAc(1) | 1208 | 527.1 | 72.0911 |
| R.ITTTPTNGQQGNSLEEVVHADQSSC[+57.021]TFDNLSPGLEYN[+1913.677]VSVYTVK.D | HexNAc(4)Hex(5)NeuAc(1) | 1208 | 513.5 | 72.8268 |
| R.ITTTPTNGQQGNSLEEVVHADQSSC[+57.021]TFDNLSPGLEYN[+1913.677]VSVYTVK.D | HexNAc(4)Hex(5)NeuAc(1) | 1208 | 601.0 | 73.8632 |
| R.ITTTPTNGQQGNSLEEVVHADQSSC[+57.021]TFDN[+2204.772]LSPGLEYNVSVYTVK.D | HexNAc(4)Hex(5)NeuAc(2) | 1208 | 165.0 | 76.1617 |
| R.ITTTPTNGQQGNSLEEVVHADQSSC[+57.021]TFDNLSPGLEYN[+2204.772]VSVYTVK.D | HexNAc(4)Hex(5)NeuAc(2) | 1208 | 434.3 | 76.4269 |
| R.ITTTPTNGQQGNSLEEVVHADQSSC[+57.021]TFDNLSPGLEYN[+1930.692]VSVYTVK.D | HexNAc(4)Hex(6)Fuc(1) | 1208 | 281.5 | 72.05 |
