# Supplementary material for: Fibronectin in cell adhesion and migration via N-glycosylation
Source: Oncotarget. 2017 Aug 7;8(41):70653–68. doi: 10.18632/oncotarget.19969 (PMC5642584; doi:10.18632/oncotarget.19969)
Supplement: Supplementary file 4 [file oncotarget-08-70653-s004.docx]

**Supplementary Table 3:** **The list of identified glycopeptides from porcine plasma fibronectin**

| **Peptide** | **Glycans** | **Starting position** | **Score** | **Scan time** |
| --- | --- | --- | --- | --- |
| R.NYT[+1241.454]DC[+57.021]TSEGR.R | HexNAc(3)Hex(3)Fuc(1) | 431 | 279.3 | 20.2571 |
| R.N[+1403.507]YTDC[+57.021]TSEGR.R | HexNAc(3)Hex(4)Fuc(1) | 431 | 298.4 | 20.2226 |
| R.N[+1403.507]YTDC[+57.021]TSEGR.R | HexNAc(3)Hex(4)Fuc(1) | 431 | 366.1 | 20.2084 |
| R.N[+1694.603]YTDC[+57.021]TSEGR.R | HexNAc(3)Hex(4)Fuc(1)NeuAc(1) | 431 | 327.3 | 20.5154 |
| R.N[+1710.598]YTDC[+57.021]TSEGR.R | HexNAc(3)Hex(5)NeuAc(1) | 431 | 103.4 | 20.5042 |
| R.N[+2018.708]YTDC[+57.021]TSEGR.R | HexNAc(3)Hex(6)Fuc(1)NeuAc(1) | 431 | 230.1 | 20.4103 |
| R.N[+2018.708]YTDC[+57.021]TSEGR.R | HexNAc(3)Hex(6)Fuc(1)NeuAc(1) | 431 | 263.6 | 20.446 |
| R.N[+1606.587]YTDC[+57.021]TSEGR.R | HexNAc(4)Hex(4)Fuc(1) | 431 | 246.0 | 19.2931 |
| R.N[+1897.682]YTDC[+57.021]TSEGRR.D | HexNAc(4)Hex(4)Fuc(1)NeuAc(1) | 431 | 113.6 | 19.0386 |
| R.N[+1897.682]YTDC[+57.021]TSEGR.R | HexNAc(4)Hex(4)Fuc(1)NeuAc(1) | 431 | 169.6 | 20.6277 |
| R.N[+1897.682]YTDC[+57.021]TSEGR.R | HexNAc(4)Hex(4)Fuc(1)NeuAc(1) | 431 | 178.8 | 20.97 |
| R.N[+1897.682]YTDC[+57.021]TSEGR.R | HexNAc(4)Hex(4)Fuc(1)NeuAc(1) | 431 | 273.9 | 20.221 |
| R.N[+1897.682]YTDC[+57.021]TSEGR.R | HexNAc(4)Hex(4)Fuc(1)NeuAc(1) | 431 | 368.9 | 20.5722 |
| R.N[+1751.624]YTDC[+57.021]TSEGR.R | HexNAc(4)Hex(4)NeuAc(1) | 431 | 191.5 | 20.4524 |
| R.N[+1751.624]YTDC[+57.021]TSEGR.R | HexNAc(4)Hex(4)NeuAc(1) | 431 | 212.9 | 20.577 |
| R.N[+1768.640]YTDC[+57.021]TSEGR.R | HexNAc(4)Hex(5)Fuc(1) | 431 | 143.1 | 19.153 |
| R.N[+1768.640]YTDC[+57.021]TSEGR.R | HexNAc(4)Hex(5)Fuc(1) | 431 | 261.6 | 19.1388 |
| R.N[+1768.640]YTDC[+57.021]TSEGR.R | HexNAc(4)Hex(5)Fuc(1) | 431 | 362.0 | 20.2806 |
| R.N[+2059.735]YTDC[+57.021]TSEGR.R | HexNAc(4)Hex(5)Fuc(1)NeuAc(1) | 431 | 147.9 | 21.7912 |
| R.N[+2059.735]YTDC[+57.021]TSEGR.R | HexNAc(4)Hex(5)Fuc(1)NeuAc(1) | 431 | 150.5 | 22.1574 |
| R.N[+2059.735]YTDC[+57.021]TSEGR.R | HexNAc(4)Hex(5)Fuc(1)NeuAc(1) | 431 | 161.4 | 19.7181 |
| R.N[+2059.735]YTDC[+57.021]TSEGR.R | HexNAc(4)Hex(5)Fuc(1)NeuAc(1) | 431 | 205.8 | 22.5383 |
| R.N[+2059.735]YTDC[+57.021]TSEGR.R | HexNAc(4)Hex(5)Fuc(1)NeuAc(1) | 431 | 213.5 | 20.7607 |
| R.N[+2059.735]YTDC[+57.021]TSEGR.R | HexNAc(4)Hex(5)Fuc(1)NeuAc(1) | 431 | 227.6 | 21.2458 |
| R.N[+2059.735]YTDC[+57.021]TSEGR.R | HexNAc(4)Hex(5)Fuc(1)NeuAc(1) | 431 | 288.7 | 20.8726 |
| R.N[+2059.735]YTDC[+57.021]TSEGR.R | HexNAc(4)Hex(5)Fuc(1)NeuAc(1) | 431 | 291.4 | 20.0463 |
| R.N[+2059.735]YTDC[+57.021]TSEGR.R | HexNAc(4)Hex(5)Fuc(1)NeuAc(1) | 431 | 298.6 | 19.8404 |
| R.N[+2059.735]YTDC[+57.021]TSEGRR.D | HexNAc(4)Hex(5)Fuc(1)NeuAc(1) | 431 | 309.8 | 18.9258 |
| R.N[+2059.735]YTDC[+57.021]TSEGR.R | HexNAc(4)Hex(5)Fuc(1)NeuAc(1) | 431 | 323.2 | 20.5264 |
| R.N[+2059.735]YTDC[+57.021]TSEGR.R | HexNAc(4)Hex(5)Fuc(1)NeuAc(1) | 431 | 329.5 | 20.3867 |
| R.N[+2059.735]YTDC[+57.021]TSEGR.R | HexNAc(4)Hex(5)Fuc(1)NeuAc(1) | 431 | 364.6 | 20.1848 |
| R.N[+2366.825]YTDC[+57.021]TSEGR.R | HexNAc(4)Hex(5)Fuc(1)NeuAc(1)NeuGc(1) | 431 | 115.0 | 22.7306 |
| R.N[+2366.825]YTDC[+57.021]TSEGR.R | HexNAc(4)Hex(5)Fuc(1)NeuAc(1)NeuGc(1) | 431 | 243.4 | 23.086 |
| R.N[+2350.830]YTDC[+57.021]TSEGR.R | HexNAc(4)Hex(5)Fuc(1)NeuAc(2) | 431 | 128.6 | 22.7959 |
| R.N[+2350.830]YTDC[+57.021]TSEGR.R | HexNAc(4)Hex(5)Fuc(1)NeuAc(2) | 431 | 229.2 | 24.2166 |
| R.N[+2350.830]YTDC[+57.021]TSEGR.R | HexNAc(4)Hex(5)Fuc(1)NeuAc(2) | 431 | 245.3 | 23.3732 |
| R.N[+2350.830]YTDC[+57.021]TSEGR.R | HexNAc(4)Hex(5)Fuc(1)NeuAc(2) | 431 | 268.0 | 22.9232 |
| R.N[+2350.830]YTDC[+57.021]TSEGR.R | HexNAc(4)Hex(5)Fuc(1)NeuAc(2) | 431 | 319.7 | 23.8527 |
| R.N[+2350.830]YTDC[+57.021]TSEGR.R | HexNAc(4)Hex(5)Fuc(1)NeuAc(2) | 431 | 349.7 | 23.1439 |
| R.N[+2350.830]YTDC[+57.021]TSEGR.R | HexNAc(4)Hex(5)Fuc(1)NeuAc(2) | 431 | 369.6 | 23.4894 |
| R.N[+2075.730]YTDC[+57.021]TSEGR.R | HexNAc(4)Hex(5)Fuc(1)NeuGc(1) | 431 | 179.3 | 20.0557 |
| R.N[+2075.730]YTDC[+57.021]TSEGR.R | HexNAc(4)Hex(5)Fuc(1)NeuGc(1) | 431 | 252.5 | 19.8918 |
| R.N[+1913.677]YTDC[+57.021]TSEGR.R | HexNAc(4)Hex(5)NeuAc(1) | 431 | 189.7 | 20.3134 |
| R.N[+1913.677]YTDC[+57.021]TSEGR.R | HexNAc(4)Hex(5)NeuAc(1) | 431 | 355.1 | 20.1927 |
| R.N[+1930.692]YTDC[+57.021]TSEGR.R | HexNAc(4)Hex(6)Fuc(1) | 431 | 125.4 | 19.2479 |
| R.N[+1930.692]YTDC[+57.021]TSEGR.R | HexNAc(4)Hex(6)Fuc(1) | 431 | 180.0 | 19.0886 |
| R.N[+2221.788]YTDC[+57.021]TSEGR.R | HexNAc(4)Hex(6)Fuc(1)NeuAc(1) | 431 | 156.9 | 19.8788 |
| R.N[+2237.783]YTDC[+57.021]TSEGR.R | HexNAc(4)Hex(6)Fuc(1)NeuGc(1) | 431 | 129.2 | 19.8468 |
| R.N[+2092.745]YTDC[+57.021]TSEGR.R | HexNAc(4)Hex(7)Fuc(1) | 431 | 247.2 | 19.0681 |
| R.DQC[+57.021]IVDDITYNVN[+1606.587]DTFHK.R | HexNAc(4)Hex(4)Fuc(1) | 517 | 184.1 | 58.5526 |
| R.DQC[+57.021]IVDDITYNVN[+1606.587]DTFHK.R | HexNAc(4)Hex(4)Fuc(1) | 517 | 238.9 | 58.4483 |
| R.DQC[+57.021]IVDDITYNVN[+1622.582]DTFHK.R | HexNAc(4)Hex(5) | 517 | 323.3 | 110.6642 |
| R.DQC[+57.021]IVDDITYNVN[+1768.640]DTFHK.R | HexNAc(4)Hex(5)Fuc(1) | 517 | 381.7 | 57.9211 |
| R.DQC[+57.021]IVDDITYNVN[+1768.640]DTFHK.R | HexNAc(4)Hex(5)Fuc(1) | 517 | 430.3 | 58.1958 |
| R.DQC[+57.021]IVDDITYNVN[+2059.735]DTFHK.R | HexNAc(4)Hex(5)Fuc(1)NeuAc(1) | 517 | 196.6 | 62.4316 |
| R.DQC[+57.021]IVDDITYNVN[+2059.735]DTFHK.R | HexNAc(4)Hex(5)Fuc(1)NeuAc(1) | 517 | 421.3 | 63.1291 |
| R.DQC[+57.021]IVDDITYNVN[+2059.735]DTFHK.R | HexNAc(4)Hex(5)Fuc(1)NeuAc(1) | 517 | 487.1 | 64.4015 |
| R.DQC[+57.021]IVDDITYNVN[+2059.735]DTFHK.R | HexNAc(4)Hex(5)Fuc(1)NeuAc(1) | 517 | 572.7 | 61.9742 |
| R.DQC[+57.021]IVDDITYNVN[+2059.735]DTFHK.R | HexNAc(4)Hex(5)Fuc(1)NeuAc(1) | 517 | 587.1 | 66.6231 |
| R.DQC[+57.021]IVDDITYNVN[+2059.735]DTFHK.R | HexNAc(4)Hex(5)Fuc(1)NeuAc(1) | 517 | 635.4 | 62.3359 |
| R.DQC[+57.021]IVDDITYNVN[+2059.735]DTFHK.R | HexNAc(4)Hex(5)Fuc(1)NeuAc(1) | 517 | 708.0 | 62.7101 |
| R.DQC[+57.021]IVDDITYNVN[+2059.735]DTFHK.R | HexNAc(4)Hex(5)Fuc(1)NeuAc(1) | 517 | 735.7 | 62.6998 |
| R.DQC[+57.021]IVDDITYNVN[+2059.735]DTFHK.R | HexNAc(4)Hex(5)Fuc(1)NeuAc(1) | 517 | 778.0 | 61.9251 |
| R.DQC[+57.021]IVDDITYNVN[+2366.825]DTFHK.R | HexNAc(4)Hex(5)Fuc(1)NeuAc(1)NeuGc(1) | 517 | 287.9 | 71.1966 |
| R.DQC[+57.021]IVDDITYNVN[+2366.825]DTFHK.R | HexNAc(4)Hex(5)Fuc(1)NeuAc(1)NeuGc(1) | 517 | 359.3 | 69.8134 |
| R.DQC[+57.021]IVDDITYNVN[+2366.825]DTFHK.R | HexNAc(4)Hex(5)Fuc(1)NeuAc(1)NeuGc(1) | 517 | 468.4 | 66.8273 |
| R.DQC[+57.021]IVDDITYNVN[+2366.825]DTFHK.R | HexNAc(4)Hex(5)Fuc(1)NeuAc(1)NeuGc(1) | 517 | 535.4 | 66.9797 |
| R.DQC[+57.021]IVDDITYNVN[+2366.825]DTFHK.R | HexNAc(4)Hex(5)Fuc(1)NeuAc(1)NeuGc(1) | 517 | 576.4 | 66.304 |
| R.DQC[+57.021]IVDDITYNVN[+2366.825]DTFHK.R | HexNAc(4)Hex(5)Fuc(1)NeuAc(1)NeuGc(1) | 517 | 600.1 | 68.1879 |
| R.DQC[+57.021]IVDDITYNVN[+2366.825]DTFHK.R | HexNAc(4)Hex(5)Fuc(1)NeuAc(1)NeuGc(1) | 517 | 706.9 | 66.3895 |
| R.DQC[+57.021]IVDDITYNVN[+2366.825]DTFHK.R | HexNAc(4)Hex(5)Fuc(1)NeuAc(1)NeuGc(1) | 517 | 720.9 | 66.4396 |
| R.DQC[+57.021]IVDDITYNVN[+2366.825]DTFHK.R | HexNAc(4)Hex(5)Fuc(1)NeuAc(1)NeuGc(1) | 517 | 723.1 | 66.7609 |
| R.DQC[+57.021]IVDDITYNVN[+2350.830]DTFHK.R | HexNAc(4)Hex(5)Fuc(1)NeuAc(2) | 517 | 30.0 | 111.7288 |
| R.DQC[+57.021]IVDDITYNVN[+2350.830]DTFHK.R | HexNAc(4)Hex(5)Fuc(1)NeuAc(2) | 517 | 61.5 | 88.1706 |
| R.DQC[+57.021]IVDDITYNVN[+2350.830]DTFHK.R | HexNAc(4)Hex(5)Fuc(1)NeuAc(2) | 517 | 102.0 | 85.9309 |
| R.DQC[+57.021]IVDDITYNVN[+2350.830]DTFHK.R | HexNAc(4)Hex(5)Fuc(1)NeuAc(2) | 517 | 140.9 | 111.0446 |
| R.DQC[+57.021]IVDDITYNVN[+2350.830]DTFHK.R | HexNAc(4)Hex(5)Fuc(1)NeuAc(2) | 517 | 182.6 | 84.2432 |
| R.DQC[+57.021]IVDDITYNVN[+2350.830]DTFHK.R | HexNAc(4)Hex(5)Fuc(1)NeuAc(2) | 517 | 220.7 | 83.8521 |
| R.DQC[+57.021]IVDDITYNVN[+2350.830]DTFHK.R | HexNAc(4)Hex(5)Fuc(1)NeuAc(2) | 517 | 261.4 | 87.2743 |
| R.DQC[+57.021]IVDDITYNVN[+2350.830]DTFHK.R | HexNAc(4)Hex(5)Fuc(1)NeuAc(2) | 517 | 334.5 | 84.184 |
| R.DQC[+57.021]IVDDITYNVN[+2350.830]DTFHK.R | HexNAc(4)Hex(5)Fuc(1)NeuAc(2) | 517 | 346.8 | 79.2924 |
| R.DQC[+57.021]IVDDITYNVN[+2350.830]DTFHK.R | HexNAc(4)Hex(5)Fuc(1)NeuAc(2) | 517 | 350.1 | 78.9082 |
| R.DQC[+57.021]IVDDITYNVN[+2350.830]DTFHK.R | HexNAc(4)Hex(5)Fuc(1)NeuAc(2) | 517 | 362.3 | 78.205 |
| R.DQC[+57.021]IVDDITYNVN[+2350.830]DTFHK.R | HexNAc(4)Hex(5)Fuc(1)NeuAc(2) | 517 | 375.2 | 79.5327 |
| R.DQC[+57.021]IVDDITYNVN[+2350.830]DTFHK.R | HexNAc(4)Hex(5)Fuc(1)NeuAc(2) | 517 | 390.7 | 80.1868 |
| R.DQC[+57.021]IVDDITYNVN[+2350.830]DTFHK.R | HexNAc(4)Hex(5)Fuc(1)NeuAc(2) | 517 | 404.6 | 69.3001 |
| R.DQC[+57.021]IVDDITYNVN[+2350.830]DTFHK.R | HexNAc(4)Hex(5)Fuc(1)NeuAc(2) | 517 | 419.0 | 71.4579 |
| R.DQC[+57.021]IVDDITYNVN[+2350.830]DTFHK.R | HexNAc(4)Hex(5)Fuc(1)NeuAc(2) | 517 | 422.6 | 78.8459 |
| R.DQC[+57.021]IVDDITYNVN[+2350.830]DTFHK.R | HexNAc(4)Hex(5)Fuc(1)NeuAc(2) | 517 | 423.6 | 79.9402 |
| R.DQC[+57.021]IVDDITYNVN[+2350.830]DTFHK.R | HexNAc(4)Hex(5)Fuc(1)NeuAc(2) | 517 | 429.9 | 77.9951 |
| R.DQC[+57.021]IVDDITYNVN[+2350.830]DTFHK.R | HexNAc(4)Hex(5)Fuc(1)NeuAc(2) | 517 | 458.9 | 76.6 |
| R.DQC[+57.021]IVDDITYNVN[+2350.830]DTFHK.R | HexNAc(4)Hex(5)Fuc(1)NeuAc(2) | 517 | 461.8 | 77.1393 |
| R.DQC[+57.021]IVDDITYNVN[+2350.830]DTFHK.R | HexNAc(4)Hex(5)Fuc(1)NeuAc(2) | 517 | 465.8 | 75.9585 |
| R.DQC[+57.021]IVDDITYNVN[+2350.830]DTFHK.R | HexNAc(4)Hex(5)Fuc(1)NeuAc(2) | 517 | 524.9 | 74.5461 |
| R.DQC[+57.021]IVDDITYNVN[+2350.830]DTFHK.R | HexNAc(4)Hex(5)Fuc(1)NeuAc(2) | 517 | 531.9 | 74.1197 |
| R.DQC[+57.021]IVDDITYNVN[+2350.830]DTFHK.R | HexNAc(4)Hex(5)Fuc(1)NeuAc(2) | 517 | 537.2 | 69.2132 |
| R.DQC[+57.021]IVDDITYNVN[+2350.830]DTFHK.R | HexNAc(4)Hex(5)Fuc(1)NeuAc(2) | 517 | 572.8 | 75.4131 |
| R.DQC[+57.021]IVDDITYNVN[+2350.830]DTFHK.R | HexNAc(4)Hex(5)Fuc(1)NeuAc(2) | 517 | 576.9 | 67.153 |
| R.DQC[+57.021]IVDDITYNVN[+2350.830]DTFHK.R | HexNAc(4)Hex(5)Fuc(1)NeuAc(2) | 517 | 584.0 | 110.6317 |
| R.DQC[+57.021]IVDDITYNVN[+2350.830]DTFHK.R | HexNAc(4)Hex(5)Fuc(1)NeuAc(2) | 517 | 586.2 | 75.5733 |
| R.DQC[+57.021]IVDDITYNVN[+2350.830]DTFHK.R | HexNAc(4)Hex(5)Fuc(1)NeuAc(2) | 517 | 588.5 | 69.0962 |
| R.DQC[+57.021]IVDDITYNVN[+2350.830]DTFHK.R | HexNAc(4)Hex(5)Fuc(1)NeuAc(2) | 517 | 601.3 | 72.9982 |
| R.DQC[+57.021]IVDDITYNVN[+2350.830]DTFHK.R | HexNAc(4)Hex(5)Fuc(1)NeuAc(2) | 517 | 609.2 | 110.6332 |
| R.DQC[+57.021]IVDDITYNVN[+2350.830]DTFHK.R | HexNAc(4)Hex(5)Fuc(1)NeuAc(2) | 517 | 630.8 | 74.6913 |
| R.DQC[+57.021]IVDDITYNVN[+2350.830]DTFHK.R | HexNAc(4)Hex(5)Fuc(1)NeuAc(2) | 517 | 637.6 | 66.3802 |
| R.DQC[+57.021]IVDDITYNVN[+2350.830]DTFHK.R | HexNAc(4)Hex(5)Fuc(1)NeuAc(2) | 517 | 655.7 | 69.7526 |
| R.DQC[+57.021]IVDDITYNVN[+2350.830]DTFHK.R | HexNAc(4)Hex(5)Fuc(1)NeuAc(2) | 517 | 662.1 | 66.328 |
| R.DQC[+57.021]IVDDITYNVN[+2350.830]DTFHK.R | HexNAc(4)Hex(5)Fuc(1)NeuAc(2) | 517 | 670.3 | 72.6128 |
| R.DQC[+57.021]IVDDITYNVN[+2350.830]DTFHK.R | HexNAc(4)Hex(5)Fuc(1)NeuAc(2) | 517 | 671.8 | 73.4939 |
| R.DQC[+57.021]IVDDITYNVN[+2350.830]DTFHK.R | HexNAc(4)Hex(5)Fuc(1)NeuAc(2) | 517 | 675.7 | 67.1719 |
| R.DQC[+57.021]IVDDITYNVN[+2350.830]DTFHK.R | HexNAc(4)Hex(5)Fuc(1)NeuAc(2) | 517 | 681.2 | 70.5447 |
| R.DQC[+57.021]IVDDITYNVN[+2350.830]DTFHK.R | HexNAc(4)Hex(5)Fuc(1)NeuAc(2) | 517 | 682.4 | 73.3253 |
| R.DQC[+57.021]IVDDITYNVN[+2350.830]DTFHK.R | HexNAc(4)Hex(5)Fuc(1)NeuAc(2) | 517 | 690.0 | 69.5852 |
| R.DQC[+57.021]IVDDITYNVN[+2350.830]DTFHK.R | HexNAc(4)Hex(5)Fuc(1)NeuAc(2) | 517 | 693.4 | 72.193 |
| R.DQC[+57.021]IVDDITYNVN[+2350.830]DTFHK.R | HexNAc(4)Hex(5)Fuc(1)NeuAc(2) | 517 | 709.5 | 67.5487 |
| R.DQC[+57.021]IVDDITYNVN[+2350.830]DTFHK.R | HexNAc(4)Hex(5)Fuc(1)NeuAc(2) | 517 | 733.0 | 71.4494 |
| R.DQC[+57.021]IVDDITYNVN[+2350.830]DTFHK.R | HexNAc(4)Hex(5)Fuc(1)NeuAc(2) | 517 | 736.5 | 67.5378 |
| R.DQC[+57.021]IVDDITYNVN[+2350.830]DTFHK.R | HexNAc(4)Hex(5)Fuc(1)NeuAc(2) | 517 | 742.8 | 68.297 |
| R.DQC[+57.021]IVDDITYNVN[+2075.730]DTFHK.R | HexNAc(4)Hex(5)Fuc(1)NeuGc(1) | 517 | 375.2 | 61.9635 |
| R.DQC[+57.021]IVDDITYNVN[+2075.730]DTFHK.R | HexNAc(4)Hex(5)Fuc(1)NeuGc(1) | 517 | 555.3 | 62.3641 |
| R.DQC[+57.021]IVDDITYNVN[+2075.730]DTFHK.R | HexNAc(4)Hex(5)Fuc(1)NeuGc(1) | 517 | 725.4 | 61.9236 |
| R.DQC[+57.021]IVDDITYNVN[+2075.730]DTFHK.R | HexNAc(4)Hex(5)Fuc(1)NeuGc(1) | 517 | 759.1 | 61.9689 |
| R.DQC[+57.021]IVDDITYNVN[+2382.820]DTFHK.R | HexNAc(4)Hex(5)Fuc(1)NeuGc(2) | 517 | 190.6 | 67.2388 |
| R.DQC[+57.021]IVDDITYNVN[+2382.820]DTFHK.R | HexNAc(4)Hex(5)Fuc(1)NeuGc(2) | 517 | 454.3 | 66.6387 |
| R.DQC[+57.021]IVDDITYNVN[+1930.692]DTFHK.R | HexNAc(4)Hex(6)Fuc(1) | 517 | 128.6 | 57.8738 |
| R.DQC[+57.021]IVDDITYNVN[+1930.692]DTFHK.R | HexNAc(4)Hex(6)Fuc(1) | 517 | 740.5 | 57.811 |
| R.DQC[+57.021]IVDDITYNVN[+2221.788]DTFHK.R | HexNAc(4)Hex(6)Fuc(1)NeuAc(1) | 517 | 437.3 | 61.8504 |
| R.DQC[+57.021]IVDDITYNVN[+2237.783]DTFHK.R | HexNAc(4)Hex(6)Fuc(1)NeuGc(1) | 517 | 193.2 | 61.9941 |
| R.DQC[+57.021]IVDDITYNVN[+2237.783]DTFHK.R | HexNAc(4)Hex(6)Fuc(1)NeuGc(1) | 517 | 244.5 | 61.6113 |
| R.DQC[+57.021]IVDDITYNVN[+2237.783]DTFHK.R | HexNAc(4)Hex(6)Fuc(1)NeuGc(1) | 517 | 349.5 | 61.8535 |
| R.DQC[+57.021]IVDDITYNVN[+2237.783]DTFHK.R | HexNAc(4)Hex(6)Fuc(1)NeuGc(1) | 517 | 541.9 | 61.5622 |
| R.DQC[+57.021]IVDDITYNVN[+2237.783]DTFHK.R | HexNAc(4)Hex(6)Fuc(1)NeuGc(1) | 517 | 637.5 | 61.755 |
| R.DQC[+57.021]IVDDITYNVN[+2237.783]DTFHK.R | HexNAc(4)Hex(6)Fuc(1)NeuGc(1) | 517 | 677.6 | 61.7327 |
| R.DQC[+57.021]IVDDITYNVN[+2092.745]DTFHK.R | HexNAc(4)Hex(7)Fuc(1) | 517 | 443.3 | 57.7929 |
| R.DQC[+57.021]IVDDITYNVN[+2424.867]DTFHK.R | HexNAc(5)Hex(6)Fuc(1)NeuAc(1) | 517 | 244.3 | 61.852 |
| R.DQC[+57.021]IVDDITYNVN[+2424.867]DTFHK.R | HexNAc(5)Hex(6)Fuc(1)NeuAc(1) | 517 | 429.7 | 61.7773 |
| R.DQC[+57.021]IVDDITYNVN[+2294.804]DTFHK.R | HexNAc(5)Hex(6)NeuGc(1) | 517 | 130.8 | 68.5452 |
| R.DQC[+57.021]IVDDITYNVN[+2352.846]DTFHK.R | HexNAc(6)Hex(7) | 517 | 30.0 | 111.1291 |
| R.DQC[+57.021]IVDDITYNVN[+2352.846]DTFHK.R | HexNAc(6)Hex(7) | 517 | 208.3 | 80.3035 |
| R.DQC[+57.021]IVDDITYNVN[+2352.846]DTFHK.R | HexNAc(6)Hex(7) | 517 | 287.8 | 71.5217 |
| R.DQC[+57.021]IVDDITYNVN[+2352.846]DTFHK.R | HexNAc(6)Hex(7) | 517 | 329.3 | 74.0771 |
| R.DQC[+57.021]IVDDITYNVN[+2352.846]DTFHK.R | HexNAc(6)Hex(7) | 517 | 409.2 | 75.0151 |
| R.DQC[+57.021]IVDDITYNVN[+2352.846]DTFHK.R | HexNAc(6)Hex(7) | 517 | 494.8 | 72.2009 |
| R.DQC[+57.021]IVDDITYNVN[+2352.846]DTFHK.R | HexNAc(6)Hex(7) | 517 | 518.5 | 70.2255 |
| R.DQC[+57.021]IVDDITYNVN[+2352.846]DTFHK.R | HexNAc(6)Hex(7) | 517 | 568.3 | 71.8053 |
| R.DQC[+57.021]IVDDITYNVN[+2352.846]DTFHK.R | HexNAc(6)Hex(7) | 517 | 638.2 | 68.5166 |
| R.DQC[+57.021]IVDDITYNVN[+2352.846]DTFHK.R | HexNAc(6)Hex(7) | 517 | 650.3 | 70.1559 |
| K.RHEEGHMLN[+1606.587]C[+57.021]TC[+57.021]FGQGR.G | HexNAc(4)Hex(4)Fuc(1) | 535 | 645.9 | 25.9438 |
| K.RHEEGHMLN[+1622.582]C[+57.021]TC[+57.021]FGQGR.G | HexNAc(4)Hex(5) | 535 | 419.0 | 26.0141 |
| K.RHEEGHMLN[+1622.582]C[+57.021]TC[+57.021]FGQGR.G | HexNAc(4)Hex(5) | 535 | 454.5 | 26.0495 |
| K.RHEEGHMLN[+1768.640]C[+57.021]TC[+57.021]FGQGR.G | HexNAc(4)Hex(5)Fuc(1) | 535 | 85.9 | 26.0995 |
| K.RHEEGHMLN[+1768.640]C[+57.021]TC[+57.021]FGQGR.G | HexNAc(4)Hex(5)Fuc(1) | 535 | 179.0 | 25.7586 |
| K.RHEEGHMLN[+1768.640]C[+57.021]TC[+57.021]FGQGR.G | HexNAc(4)Hex(5)Fuc(1) | 535 | 216.4 | 25.9927 |
| K.RHEEGHMLN[+1768.640]C[+57.021]TC[+57.021]FGQGR.G | HexNAc(4)Hex(5)Fuc(1) | 535 | 378.2 | 25.9203 |
| K.RHEEGHMLN[+1768.640]C[+57.021]TC[+57.021]FGQGR.G | HexNAc(4)Hex(5)Fuc(1) | 535 | 647.8 | 25.7329 |
| K.RHEEGHMLN[+2059.735]C[+57.021]TC[+57.021]FGQGR.G | HexNAc(4)Hex(5)Fuc(1)NeuAc(1) | 535 | 30.0 | 26.9523 |
| K.RHEEGHMLN[+2059.735]C[+57.021]TC[+57.021]FGQGR.G | HexNAc(4)Hex(5)Fuc(1)NeuAc(1) | 535 | 170.7 | 27.7253 |
| K.RHEEGHMLN[+2059.735]C[+57.021]TC[+57.021]FGQGR.G | HexNAc(4)Hex(5)Fuc(1)NeuAc(1) | 535 | 181.9 | 29.096 |
| K.RHEEGHMLN[+2059.735]C[+57.021]TC[+57.021]FGQGR.G | HexNAc(4)Hex(5)Fuc(1)NeuAc(1) | 535 | 184.8 | 27.0635 |
| K.RHEEGHMLN[+2059.735]C[+57.021]TC[+57.021]FGQGR.G | HexNAc(4)Hex(5)Fuc(1)NeuAc(1) | 535 | 223.0 | 28.8162 |
| K.RHEEGHMLN[+2059.735]C[+57.021]TC[+57.021]FGQGR.G | HexNAc(4)Hex(5)Fuc(1)NeuAc(1) | 535 | 279.0 | 28.8627 |
| K.RHEEGHMLN[+2059.735]C[+57.021]TC[+57.021]FGQGR.G | HexNAc(4)Hex(5)Fuc(1)NeuAc(1) | 535 | 312.5 | 27.0982 |
| K.RHEEGHMLN[+2059.735]C[+57.021]TC[+57.021]FGQGR.G | HexNAc(4)Hex(5)Fuc(1)NeuAc(1) | 535 | 342.2 | 29.4787 |
| K.RHEEGHMLN[+2059.735]C[+57.021]TC[+57.021]FGQGR.G | HexNAc(4)Hex(5)Fuc(1)NeuAc(1) | 535 | 357.7 | 26.9877 |
| K.RHEEGHMLN[+2059.735]C[+57.021]TC[+57.021]FGQGR.G | HexNAc(4)Hex(5)Fuc(1)NeuAc(1) | 535 | 412.5 | 27.4442 |
| K.RHEEGHMLN[+2366.825]C[+57.021]TC[+57.021]FGQGR.G | HexNAc(4)Hex(5)Fuc(1)NeuAc(1)NeuGc(1) | 535 | 70.9 | 30.6027 |
| K.RHEEGHMLN[+2366.825]C[+57.021]TC[+57.021]FGQGR.G | HexNAc(4)Hex(5)Fuc(1)NeuAc(1)NeuGc(1) | 535 | 250.5 | 30.9783 |
| K.RHEEGHMLN[+2366.825]C[+57.021]TC[+57.021]FGQGR.G | HexNAc(4)Hex(5)Fuc(1)NeuAc(1)NeuGc(1) | 535 | 398.1 | 30.6287 |
| K.RHEEGHMLN[+2350.830]C[+57.021]TC[+57.021]FGQGR.G | HexNAc(4)Hex(5)Fuc(1)NeuAc(2) | 535 | 181.4 | 30.6957 |
| K.RHEEGHMLN[+2350.830]C[+57.021]TC[+57.021]FGQGR.G | HexNAc(4)Hex(5)Fuc(1)NeuAc(2) | 535 | 265.3 | 31.1149 |
| K.RHEEGHMLN[+2350.830]C[+57.021]TC[+57.021]FGQGR.G | HexNAc(4)Hex(5)Fuc(1)NeuAc(2) | 535 | 311.7 | 31.0521 |
| K.RHEEGHMLN[+2350.830]C[+57.021]TC[+57.021]FGQGR.G | HexNAc(4)Hex(5)Fuc(1)NeuAc(2) | 535 | 314.8 | 29.8257 |
| K.RHEEGHMLN[+2350.830]C[+57.021]TC[+57.021]FGQGR.G | HexNAc(4)Hex(5)Fuc(1)NeuAc(2) | 535 | 318.8 | 29.8056 |
| K.RHEEGHMLN[+2350.830]C[+57.021]TC[+57.021]FGQGR.G | HexNAc(4)Hex(5)Fuc(1)NeuAc(2) | 535 | 445.4 | 31.2685 |
| K.RHEEGHMLN[+2075.730]C[+57.021]TC[+57.021]FGQGR.G | HexNAc(4)Hex(5)Fuc(1)NeuGc(1) | 535 | 294.4 | 26.9444 |
| K.RHEEGHMLN[+1913.677]C[+57.021]TC[+57.021]FGQGR.G | HexNAc(4)Hex(5)NeuAc(1) | 535 | 138.4 | 27.8827 |
| K.RHEEGHMLN[+2220.767]C[+57.021]TC[+57.021]FGQGR.G | HexNAc(4)Hex(5)NeuAc(1)NeuGc(1) | 535 | 256.1 | 31.1076 |
| K.RHEEGHMLN[+2204.772]C[+57.021]TC[+57.021]FGQGR.G | HexNAc(4)Hex(5)NeuAc(2) | 535 | 130.1 | 31.1321 |
| K.RHEEGHMLN[+2204.772]C[+57.021]TC[+57.021]FGQGR.G | HexNAc(4)Hex(5)NeuAc(2) | 535 | 495.7 | 31.1032 |
| K.RHEEGHMLN[+2204.772]C[+57.021]TC[+57.021]FGQGR.G | HexNAc(4)Hex(5)NeuAc(2) | 535 | 566.6 | 31.0959 |
| K.RHEEGHMLN[+2204.772]C[+57.021]TC[+57.021]FGQGR.G | HexNAc(4)Hex(5)NeuAc(2) | 535 | 586.4 | 31.2908 |
| K.RHEEGHMLN[+1930.692]C[+57.021]TC[+57.021]FGQGR.G | HexNAc(4)Hex(6)Fuc(1) | 535 | 204.6 | 25.6849 |
| K.RHEEGHMLN[+1930.692]C[+57.021]TC[+57.021]FGQGR.G | HexNAc(4)Hex(6)Fuc(1) | 535 | 338.0 | 25.6663 |
| K.RHEEGHMLN[+1930.692]C[+57.021]TC[+57.021]FGQGR.G | HexNAc(4)Hex(6)Fuc(1) | 535 | 553.4 | 25.6691 |
| K.RHEEGHMLN[+1930.692]C[+57.021]TC[+57.021]FGQGR.G | HexNAc(4)Hex(6)Fuc(1) | 535 | 616.7 | 25.6706 |
| K.RHEEGHMLN[+2221.788]C[+57.021]TC[+57.021]FGQGR.G | HexNAc(4)Hex(6)Fuc(1)NeuAc(1) | 535 | 30.0 | 26.9133 |
| K.RHEEGHMLN[+2092.745]C[+57.021]TC[+57.021]FGQGR.G | HexNAc(4)Hex(7)Fuc(1) | 535 | 160.4 | 25.5522 |
| K.RHEEGHMLN[+2092.745]C[+57.021]TC[+57.021]FGQGR.G | HexNAc(4)Hex(7)Fuc(1) | 535 | 568.4 | 25.6802 |
| K.RHEEGHMLN[+2092.745]C[+57.021]TC[+57.021]FGQGR.G | HexNAc(4)Hex(7)Fuc(1) | 535 | 611.0 | 25.5569 |
| K.RHEEGHMLN[+2237.783]C[+57.021]TC[+57.021]FGQGR.G | HexNAc(4)Hex(7)NeuAc(1) | 535 | 146.2 | 36.7356 |
| K.RHEEGHMLN[+2237.783]C[+57.021]TC[+57.021]FGQGR.G | HexNAc(4)Hex(7)NeuAc(1) | 535 | 193.2 | 36.7503 |
| K.RHEEGHMLN[+2237.783]C[+57.021]TC[+57.021]FGQGR.G | HexNAc(4)Hex(7)NeuAc(1) | 535 | 212.7 | 36.7732 |
| R.HEEGHMLN[+1216.423]C[+57.021]TC[+57.021]FGQGR.G | HexNAc(2)Hex(5) | 536 | 766.2 | 29.647 |
| R.HEEGHMLN[+1606.587]C[+57.021]TC[+57.021]FGQGR.G | HexNAc(4)Hex(4)Fuc(1) | 536 | 444.8 | 29.0418 |
| R.HEEGHMLN[+1606.587]C[+57.021]TC[+57.021]FGQGR.G | HexNAc(4)Hex(4)Fuc(1) | 536 | 780.8 | 29.1037 |
| R.HEEGHMLN[+1897.682]C[+57.021]TC[+57.021]FGQGR.G | HexNAc(4)Hex(4)Fuc(1)NeuAc(1) | 536 | 447.0 | 31.3737 |
| R.HEEGHMLN[+1897.682]C[+57.021]TC[+57.021]FGQGR.G | HexNAc(4)Hex(4)Fuc(1)NeuAc(1) | 536 | 735.3 | 31.4044 |
| R.HEEGHMLN[+1622.582]C[+57.021]TC[+57.021]FGQGR.G | HexNAc(4)Hex(5) | 536 | 228.2 | 110.8826 |
| R.HEEGHMLN[+1622.582]C[+57.021]TC[+57.021]FGQGR.G | HexNAc(4)Hex(5) | 536 | 683.6 | 3.0281 |
| R.HEEGHMLN[+1768.640]C[+57.021]TC[+57.021]FGQGR.G | HexNAc(4)Hex(5)Fuc(1) | 536 | 609.1 | 29.0166 |
| R.HEEGHMLN[+1768.640]C[+57.021]TC[+57.021]FGQGR.G | HexNAc(4)Hex(5)Fuc(1) | 536 | 722.5 | 28.9381 |
| R.HEEGHMLN[+2059.735]C[+57.021]TC[+57.021]FGQGR.G | HexNAc(4)Hex(5)Fuc(1)NeuAc(1) | 536 | 368.9 | 31.0413 |
| R.HEEGHMLN[+2059.735]C[+57.021]TC[+57.021]FGQGR.G | HexNAc(4)Hex(5)Fuc(1)NeuAc(1) | 536 | 465.2 | 32.0741 |
| R.HEEGHMLN[+2059.735]C[+57.021]TC[+57.021]FGQGR.G | HexNAc(4)Hex(5)Fuc(1)NeuAc(1) | 536 | 591.7 | 31.3168 |
| R.HEEGHMLN[+2059.735]C[+57.021]TC[+57.021]FGQGR.G | HexNAc(4)Hex(5)Fuc(1)NeuAc(1) | 536 | 700.7 | 34.2694 |
| R.HEEGHMLN[+2059.735]C[+57.021]TC[+57.021]FGQGR.G | HexNAc(4)Hex(5)Fuc(1)NeuAc(1) | 536 | 854.3 | 31.0339 |
| R.HEEGHMLN[+2059.735]C[+57.021]TC[+57.021]FGQGR.G | HexNAc(4)Hex(5)Fuc(1)NeuAc(1) | 536 | 878.0 | 31.0426 |
| R.HEEGHMLN[+2366.825]C[+57.021]TC[+57.021]FGQGR.G | HexNAc(4)Hex(5)Fuc(1)NeuAc(1)NeuGc(1) | 536 | 70.4 | 34.5368 |
| R.HEEGHMLN[+2366.825]C[+57.021]TC[+57.021]FGQGR.G | HexNAc(4)Hex(5)Fuc(1)NeuAc(1)NeuGc(1) | 536 | 298.7 | 29.1364 |
| R.HEEGHMLN[+2366.825]C[+57.021]TC[+57.021]FGQGR.G | HexNAc(4)Hex(5)Fuc(1)NeuAc(1)NeuGc(1) | 536 | 735.7 | 34.1572 |
| R.HEEGHMLN[+2366.825]C[+57.021]TC[+57.021]FGQGR.G | HexNAc(4)Hex(5)Fuc(1)NeuAc(1)NeuGc(1) | 536 | 848.0 | 34.1057 |
| R.HEEGHMLN[+2350.830]C[+57.021]TC[+57.021]FGQGR.G | HexNAc(4)Hex(5)Fuc(1)NeuAc(2) | 536 | 464.4 | 34.9442 |
| R.HEEGHMLN[+2350.830]C[+57.021]TC[+57.021]FGQGR.G | HexNAc(4)Hex(5)Fuc(1)NeuAc(2) | 536 | 695.4 | 34.5574 |
| R.HEEGHMLN[+2350.830]C[+57.021]TC[+57.021]FGQGR.G | HexNAc(4)Hex(5)Fuc(1)NeuAc(2) | 536 | 709.5 | 34.1584 |
| R.HEEGHMLN[+2350.830]C[+57.021]TC[+57.021]FGQGR.G | HexNAc(4)Hex(5)Fuc(1)NeuAc(2) | 536 | 723.2 | 34.1713 |
| R.HEEGHMLN[+2350.830]C[+57.021]TC[+57.021]FGQGR.G | HexNAc(4)Hex(5)Fuc(1)NeuAc(2) | 536 | 829.7 | 34.1496 |
| R.HEEGHMLN[+2075.730]C[+57.021]TC[+57.021]FGQGR.G | HexNAc(4)Hex(5)Fuc(1)NeuGc(1) | 536 | 549.1 | 31.0226 |
| R.HEEGHMLN[+2075.730]C[+57.021]TC[+57.021]FGQGR.G | HexNAc(4)Hex(5)Fuc(1)NeuGc(1) | 536 | 785.3 | 30.9991 |
| R.HEEGHMLN[+2382.820]C[+57.021]TC[+57.021]FGQGR.G | HexNAc(4)Hex(5)Fuc(1)NeuGc(2) | 536 | 635.7 | 34.0599 |
| R.HEEGHMLN[+1913.677]C[+57.021]TC[+57.021]FGQGR.G | HexNAc(4)Hex(5)NeuAc(1) | 536 | 840.2 | 31.337 |
| R.HEEGHMLN[+2220.767]C[+57.021]TC[+57.021]FGQGR.G | HexNAc(4)Hex(5)NeuAc(1)NeuGc(1) | 536 | 603.7 | 34.7313 |
| R.HEEGHMLN[+2204.772]C[+57.021]TC[+57.021]FGQGR.G | HexNAc(4)Hex(5)NeuAc(2) | 536 | 469.2 | 35.19 |
| R.HEEGHMLN[+2204.772]C[+57.021]TC[+57.021]FGQGR.G | HexNAc(4)Hex(5)NeuAc(2) | 536 | 643.0 | 34.8166 |
| R.HEEGHMLN[+2204.772]C[+57.021]TC[+57.021]FGQGR.G | HexNAc(4)Hex(5)NeuAc(2) | 536 | 804.5 | 34.8408 |
| R.HEEGHMLN[+2236.762]C[+57.021]TC[+57.021]FGQGR.G | HexNAc(4)Hex(5)NeuGc(2) | 536 | 214.7 | 40.6391 |
| R.HEEGHMLN[+2236.762]C[+57.021]TC[+57.021]FGQGR.G | HexNAc(4)Hex(5)NeuGc(2) | 536 | 253.8 | 40.6405 |
| R.HEEGHMLN[+1930.692]C[+57.021]TC[+57.021]FGQGR.G | HexNAc(4)Hex(6)Fuc(1) | 536 | 144.0 | 27.7129 |
| R.HEEGHMLN[+1930.692]C[+57.021]TC[+57.021]FGQGR.G | HexNAc(4)Hex(6)Fuc(1) | 536 | 168.9 | 28.0847 |
| R.HEEGHMLN[+1930.692]C[+57.021]TC[+57.021]FGQGR.G | HexNAc(4)Hex(6)Fuc(1) | 536 | 281.9 | 27.6905 |
| R.HEEGHMLN[+1930.692]C[+57.021]TC[+57.021]FGQGR.G | HexNAc(4)Hex(6)Fuc(1) | 536 | 646.4 | 27.7237 |
| R.HEEGHMLN[+2075.730]C[+57.021]TC[+57.021]FGQGR.G | HexNAc(4)Hex(6)NeuAc(1) | 536 | 30.0 | 25.6489 |
| R.HEEGHMLN[+2075.730]C[+57.021]TC[+57.021]FGQGR.G | HexNAc(4)Hex(6)NeuAc(1) | 536 | 69.5 | 25.7524 |
| R.HEEGHMLN[+2075.730]C[+57.021]TC[+57.021]FGQGR.G | HexNAc(4)Hex(6)NeuAc(1) | 536 | 104.7 | 30.938 |
| R.HEEGHMLN[+2075.730]C[+57.021]TC[+57.021]FGQGR.G | HexNAc(4)Hex(6)NeuAc(1) | 536 | 105.7 | 25.7539 |
| R.HEEGHMLN[+1946.687]C[+57.021]TC[+57.021]FGQGR.G | HexNAc(4)Hex(7) | 536 | 115.2 | 37.023 |
| R.HEEGHMLN[+2092.745]C[+57.021]TC[+57.021]FGQGR.G | HexNAc(4)Hex(7)Fuc(1) | 536 | 232.2 | 27.7439 |
| R.HEEGHMLN[+2092.745]C[+57.021]TC[+57.021]FGQGR.G | HexNAc(4)Hex(7)Fuc(1) | 536 | 436.0 | 27.2389 |
| R.HEEGHMLN[+2092.745]C[+57.021]TC[+57.021]FGQGR.G | HexNAc(4)Hex(7)Fuc(1) | 536 | 476.8 | 27.2161 |
| R.HEEGHMLN[+2262.814]C[+57.021]TC[+57.021]FGQGR.G | HexNAc(5)Hex(5)Fuc(1)NeuAc(1) | 536 | 346.4 | 38.1409 |
| R.HEEGHMLN[+2295.824]C[+57.021]TC[+57.021]FGQGR.G | HexNAc(5)Hex(7)Fuc(1) | 536 | 101.4 | 38.0106 |
| K.LDAPTNLQFIN[+2059.735]ETDSTVMVTWTPPR.A | HexNAc(4)Hex(5)Fuc(1)NeuAc(1) | 998 | 500.6 | 78.2713 |
| K.LDAPTNLQFIN[+2059.735]ETDSTVMVTWTPPR.A | HexNAc(4)Hex(5)Fuc(1)NeuAc(1) | 998 | 592.2 | 78.4611 |
| K.LDAPTNLQFIN[+2366.825]ETDSTVMVTWTPPR.A | HexNAc(4)Hex(5)Fuc(1)NeuAc(1)NeuGc(1) | 998 | 184.0 | 86.7903 |
| K.LDAPTNLQFIN[+2366.825]ETDSTVMVTWTPPR.A | HexNAc(4)Hex(5)Fuc(1)NeuAc(1)NeuGc(1) | 998 | 191.9 | 110.6014 |
| K.LDAPTNLQFIN[+2366.825]ETDSTVMVTWTPPR.A | HexNAc(4)Hex(5)Fuc(1)NeuAc(1)NeuGc(1) | 998 | 244.1 | 86.8685 |
| K.LDAPTNLQFIN[+2350.830]ETDSTVMVTWTPPR.A | HexNAc(4)Hex(5)Fuc(1)NeuAc(2) | 998 | 262.3 | 110.762 |
| K.LDAPTNLQFIN[+2350.830]ETDSTVMVTWTPPR.A | HexNAc(4)Hex(5)Fuc(1)NeuAc(2) | 998 | 582.6 | 83.4173 |
| K.LDAPTNLQFIN[+2350.830]ETDSTVMVTWTPPR.A | HexNAc(4)Hex(5)Fuc(1)NeuAc(2) | 998 | 591.8 | 86.5091 |
| K.LDAPTNLQFIN[+2350.830]ETDSTVMVTWTPPR.A | HexNAc(4)Hex(5)Fuc(1)NeuAc(2) | 998 | 644.5 | 83.8053 |
| K.LDAPTNLQFIN[+2350.830]ETDSTVMVTWTPPR.A | HexNAc(4)Hex(5)Fuc(1)NeuAc(2) | 998 | 670.0 | 83.0602 |
| K.LDAPTNLQFIN[+2221.788]ETDSTVMVTWTPPR.A | HexNAc(4)Hex(6)Fuc(1)NeuAc(1) | 998 | 351.8 | 78.2432 |
| K.LDAPTNLQFIN[+2221.788]ETDSTVMVTWTPPR.A | HexNAc(4)Hex(6)Fuc(1)NeuAc(1) | 998 | 518.1 | 77.9796 |
| K.LDAPTNLQFIN[+2237.783]ETDSTVMVTWTPPR.A | HexNAc(4)Hex(6)Fuc(1)NeuGc(1) | 998 | 435.8 | 78.0282 |
| K.LDAPTNLQFIN[+2075.730]ETDSTVMVTWTPPR.A | HexNAc(4)Hex(6)NeuAc(1) | 998 | 408.5 | 78.0805 |
| K.LDAPTNLQFIN[+2352.846]ETDSTVMVTWTPPR.A | HexNAc(6)Hex(7) | 998 | 444.8 | 86.2041 |
| R.ITTTPTNGQQGYSLEEVVHADQSSC[+57.021]TFEN[+1913.677]LSPGLEYNVSVYTVK.N | HexNAc(4)Hex(5)NeuAc(1) | 1209 | 172.9 | 79.8359 |
| R.ITTTPTNGQQGYSLEEVVHADQSSC[+57.021]TFEN[+1913.677]LSPGLEYNVSVYTVK.N | HexNAc(4)Hex(5)NeuAc(1) | 1209 | 267.4 | 75.214 |
| R.ITTTPTNGQQGYSLEEVVHADQSSC[+57.021]TFENLSPGLEYN[+1913.677]VSVYTVK.N | HexNAc(4)Hex(5)NeuAc(1) | 1209 | 308.6 | 75.4103 |
| R.ITTTPTNGQQGYSLEEVVHADQSSC[+57.021]TFENLSPGLEYN[+1913.677]VSVYTVK.N | HexNAc(4)Hex(5)NeuAc(1) | 1209 | 396.2 | 77.6306 |
| R.ITTTPTNGQQGYSLEEVVHADQSSC[+57.021]TFENLSPGLEYN[+1913.677]VSVYTVK.N | HexNAc(4)Hex(5)NeuAc(1) | 1209 | 498.4 | 75.4446 |
| R.ITTTPTNGQQGYSLEEVVHADQSSC[+57.021]TFENLSPGLEYN[+2204.772]VSVYTVK.N | HexNAc(4)Hex(5)NeuAc(2) | 1209 | 451.5 | 80.8279 |
| R.ITTTPTNGQQGYSLEEVVHADQSSC[+57.021]TFEN[+1930.692]LSPGLEYNVSVYTVK.N | HexNAc(4)Hex(6)Fuc(1) | 1209 | 218.8 | 74.7836 |
| R.ITTTPTNGQQGYSLEEVVHADQSSC[+57.021]TFEN[+2221.788]LSPGLEYNVSVYTVK.N | HexNAc(4)Hex(6)Fuc(1)NeuAc(1) | 1209 | 135.1 | 81.5648 |
| R.GDVDHHLYPHVLGLNPN[+2059.735]ASTGQEALSQTTISWTPFQESSEYIISC[+57.021]HPVGIDEEPLQFR.V | HexNAc(4)Hex(5)Fuc(1)NeuAc(1) | 2184 | 936.9 | 82.3798 |
| R.GDVDHHLYPHVLGLNPN[+2366.825]ASTGQEALSQTTISWTPFQESSEYIISC[+57.021]HPVGIDEEPLQFR.V | HexNAc(4)Hex(5)Fuc(1)NeuAc(1)NeuGc(1) | 2184 | 985.8 | 85.1559 |
| R.GDVDHHLYPHVLGLNPN[+2350.830]ASTGQEALSQTTISWTPFQESSEYIISC[+57.021]HPVGIDEEPLQFR.V | HexNAc(4)Hex(5)Fuc(1)NeuAc(2) | 2184 | 893.7 | 86.2219 |
| R.GDVDHHLYPHVLGLNPN[+2352.846]ASTGQEALSQTTISWTPFQESSEYIISC[+57.021]HPVGIDEEPLQFR.V | HexNAc(6)Hex(7) | 2184 | 792.2 | 86.5595 |
| R.GDVDHHLYPHVLGLNPN[+2352.846]ASTGQEALSQTTISWTPFQESSEYIISC[+57.021]HPVGIDEEPLQFR.V | HexNAc(6)Hex(7) | 2184 | 927.1 | 110.8565 |
| R.GDVDHHLYPHVLGLNPN[+2352.846]ASTGQEALSQTTISWTPFQESSEYIISC[+57.021]HPVGIDEEPLQFR.V | HexNAc(6)Hex(7) | 2184 | 1025.6 | 85.1796 |
| R.GDVDHHLYPHVLGLNPN[+3009.074]ASTGQEALSQTTISWTPFQESSEYIISC[+57.021]HPVGIDEEPLQFR.V | HexNAc(7)Hex(8)NeuAc(1) | 2184 | 362.6 | 86.9759 |
